# Current Status of Mesenchymal Stem/Stromal Cells for Treatment of Neurological Diseases

**DOI:** 10.3389/fnmol.2022.883378

**Published:** 2022-06-16

**Authors:** Milena B. P. Soares, Renata G. J. Gonçalves, Juliana F. Vasques, Almir J. da Silva-Junior, Fernanda Gubert, Girlaine Café Santos, Thaís Alves de Santana, Gabriela Louise Almeida Sampaio, Daniela Nascimento Silva, Massimo Dominici, Rosalia Mendez-Otero

**Affiliations:** ^1^Laboratório de Engenharia Tecidual e Imunofarmacologia, Instituto Gonçalo Moniz, Fundação Oswaldo Cruz (IGM-FIOCRUZ/BA), Salvador, Brazil; ^2^Instituto SENAI de Sistemas Avançados de Saúde (CIMATEC ISI-SAS), Centro Universitário SENAI/CIMATEC, Salvador, Brazil; ^3^Laboratório de Neurobiologia Celular e Molecular, Instituto de Biofísica Carlos Chagas Filho, Universidade Federal do Rio de Janeiro, Rio de Janeiro, Brazil; ^4^Programa Redes de Pesquisa em Saúde no Estado do Rio de Janeiro, Rio de Janeiro, Brazil; ^5^Instituto de Ciências Biomédicas, Universidade Federal do Rio de Janeiro, Rio de Janeiro, Brazil; ^6^Programa Redes de Pesquisa em Nanotecnologia no Estado do Rio de Janeiro, Rio de Janeiro, Brazil; ^7^Department of Laboratory Medicine, Karolinska Institute, Stockholm, Sweden; ^8^Laboratory of Cellular Therapy, Division of Oncology, University of Modena and Reggio Emilia (UNIMORE), Modena, Italy

**Keywords:** mesenchymal stem cells, extracellular vesicles, regenerative medicine, cell therapy, neurological diseases, neuroprotection

## Abstract

Neurological disorders include a wide spectrum of clinical conditions affecting the central and peripheral nervous systems. For these conditions, which affect hundreds of millions of people worldwide, generally limited or no treatments are available, and cell-based therapies have been intensively investigated in preclinical and clinical studies. Among the available cell types, mesenchymal stem/stromal cells (MSCs) have been widely studied but as yet no cell-based treatment exists for neurological disease. We review current knowledge of the therapeutic potential of MSC-based therapies for neurological diseases, as well as possible mechanisms of action that may be explored to hasten the development of new and effective treatments. We also discuss the challenges for culture conditions, quality control, and the development of potency tests, aiming to generate more efficient cell therapy products for neurological disorders.

## Introduction

Mesenchymal stem cells (MSCs) are among the major cell types used in regenerative medicine and represent a promising therapeutic tool for several presently incurable neurological disorders. First found in bone marrow (Friedenstein et al., 1970), MSCs can be isolated from almost any adult tissue, including fat, peripheral blood, muscle, skin, and teeth, in addition to birth-associated tissues such as umbilical cord (Wharton’s jelly and cord blood), amnion, and placenta (Berebichez-Fridman and Montero-Olvera, 2018). MSCs comprise a heterogeneous population of fibroblast-like multipotent and self-renewing cells. Regardless of the source or harvest and expansion methods, MSCs must meet three minimum criteria to ascertain their equivalence and stemness: (I) plastic adherence under standard culture conditions; (II) expression of CD105, CD73, and CD90, and lack of expression of CD45, CD34, CD14/CD11b, CD79α/CD19, and HLA-DR surface markers; and (III) ability to differentiate into osteoblasts, adipocytes, and chondroblasts (Dominici et al., 2006).

Despite these common defining characteristics, MSCs have source-dependent differences that influence their applicability. Currently, bone marrow (BM) is the most widely used source of MSCs for clinical trials, followed by the umbilical cord (UC), and adipose tissue (AT; Kabat et al., 2020). BM-MSCs have long been considered the gold standard in cell therapy, with very well-characterized properties. They are easily obtained by BM aspiration from iliac crests, allowing autologous transplantation, which reduces the risk of immunological rejection. However, the harvesting procedure, in addition to being painful, has a low cell yield that declines with increasing donor age, as do the cell lifespan, proliferative capacity, and differentiation potential (Zaim et al., 2012). In recent years, AT has been reported to be a richer and more practical source of autologous MSCs in terms of availability, abundance, and accessibility compared to BM, but the sampling procedure is technically invasive (Chu et al., 2019). Birth-related tissues such as UC have great advantages over other sources, as they can be readily collected with no pain or risk to either mother or child and are usually discarded. MSCs derived from these tissues are considered more primitive according to the expression profile of cell surface markers (Conconi et al., 2011) and have intermediate features between embryonic and adult stem cells, with multilineage differentiation potential, rapid proliferation rate, low senescence, and hypoimmunogenicity, which allows safe allogeneic transplantation (El Omar et al., 2014; Nagamura-Inoue and Mukai, 2016). Furthermore, UC tissue provides large quantities of harvestable MSCs, which can be long-term cryopreserved for future cultivation (Vangsness et al., 2015).

**Figure 1:**
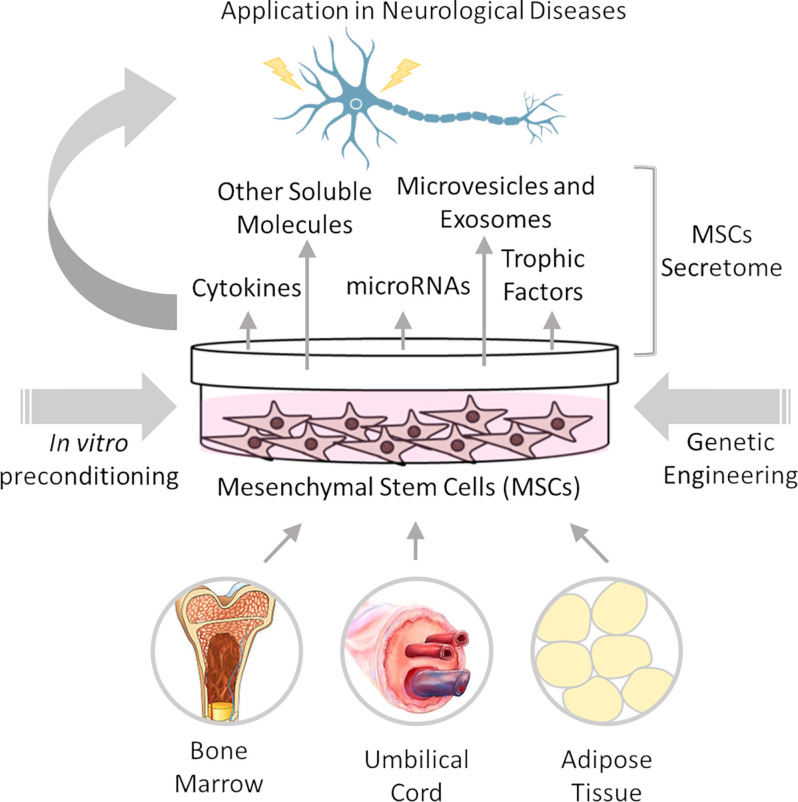
Mesenchymal stem cell therapy for neurological diseases. Mesenchymal stem/stromal cells (MSCs) can be isolated from several adult and perinatal tissues, including bone marrow, umbilical cord, and adipose tissue. MSC neuroprotective actions are based on their paracrine action through secretion of cytokines, trophic factors, and microRNAs, among other molecules, which are released directly into the extracellular space or packaged in microvesicles and exosomes. MSC efficacy can be improved *in vitro* prior to transplantation, by different preconditioning methods and/or genetic engineering to increase the production or release of specific factors.

The therapeutic potential of MSCs is attributed to their homing property, multilineage differentiation, and paracrine function. MSCs can migrate toward injured tissues, engraft, and differentiate into functional cells (Fu et al., 2019). However, rather than cellular replacement, MSCs contribute to tissue repair mainly by the paracrine action of their secretome, which comprises a wide range of immunomodulatory, angiogenic, antiapoptotic, and growth factors, supporting cell survival and tissue regeneration (Teixeira and Salgado, 2020). These features of MSCs make them promising for the treatment of neurological diseases by modulating the inflammatory and inhibitory milieu of injured/degenerating nervous tissue (Figure 1).

In this review, we discuss the methods currently employed to foster the development of more efficient cell therapy products, and also provide an overview of current research on therapeutic applications of MSCs in neurological disorders.

## MSC-Derived Exosomes and Extracellular Vesicles (Evs)

Although the transplantation of MCSs has yielded promising results in several preclinical and clinical studies, the risks of transplantation of cells that are extensively manipulated during culturing, transport, and storage persist. Furthermore, little is known about the long-term behavior of transplanted cells. The use of cell-derived products that reproduce the effects of cell therapy could allay these concerns. Thus, extracellular vesicles (Evs) derived from MSCs emerge as an alternative for therapy. To date, 74 studies related to Evs are reported as completed, are listed in clinicaltrials.org, and have posted their results and shown the safety of using Evs (NCT04281901, NCT04491240, NCT04491240; Shi et al., 2021). Evs are bilayer lipid-coated structures containing intracellular contents that can be classified by their origin and size. Exosomes are vesicles 40–150 nm in diameter that are generated by endosomes; microvesicles are vesicles 100–1,000 nm in diameter, generated from the budding of the plasma membrane (Lindoso et al., 2017). Other forms of Evs are apoptotic bodies and excretion vesicles, but in the present context of MSC mechanisms, we will refer to both exosomes and microvesicles as Evs. These Evs carry proteins, bioactive lipids, and coding and non-coding RNA and can modify the physiology of the cell target. As MSCs are very weakly immunogenic, their Evs also have low immunogenicity, which makes them safe for transplantation (Zhu et al., 2017; Montaner-Tarbes et al., 2018; Saleh et al., 2019; Rodrigues et al., 2021).

MSC-derived Evs can be identified by their affinity to certain molecules. Thus, exosomes have an affinity to cholera toxin B, which binds to the GM1 ganglioside, while annexin V and shiga toxin B bind to phosphatidylserine and globotriaosylceramide, and their biogenesis is related to the cytoplasm and nucleus, respectively (Lai and Lim, 2019). The lipid composition of Evs can define their origins and activity. The concentration of the lipid dilysocardiolipin can affect the delivery activity of Evs (Haraszti et al., 2019). Other lipids include ceramides, sphingomyelins, phosphatidylcholines, phosphatidylethanolamines, phosphatidylserines, phosphatidylinositols, eicosanoids, and cholesterol (Le Saux et al., 2020). Moreover, Evs can carry lipid mediators with immunomodulatory effects, such as prostaglandin E2 and resolvins (Holopainen et al., 2019).

### Proteins Related to MSC-Derived Evs

Proteins associated with MSC-derived Evs include antigens (e.g., CD9, CD63, and CD81), adhesion molecules, and surface receptors (e.g., PDGFRB, EGFR, and PLAUR; Kim H. S. et al., 2012). Adhesion molecules such as the RAB family are important to regulate the docking and fusion of Evs (Kim H. S. et al., 2012). MSC-derived Evs carry proteins that can sustain cellular homeostasis. In addition to typically extracellular proteins and membrane-associated proteins, Evs can transport typically intracellular proteins or even organelles, such as mitochondria (Morrison et al., 2017; Zhang Z. et al., 2020). Antioxidant enzymes such as catalase can be found in BM (Godoy et al., 2017) and Wharton’s jelly (WJ), and MSC-derived Evs have been shown to prevent oxidative stress in hippocampal neurons (Bodart-Santos et al., 2019; Puig-Pijuan et al., 2020). Inflammation can be modulated by EV-associated proteins such as CD73, TGF-β, and PTX3 (Alvarez et al., 2018; Crain et al., 2019; Kim et al., 2020). Furthermore, proteins found in AT-MSC-derived Evs are related to the MAPK, VEGF, and Jak-Stat pathways (Xing et al., 2020).

The protein composition of MSC-derived Evs can be modified by their culture conditions. Human BM-MSCs cultured with 1% O_2_ in a serum-deprived medium produced Evs with elevated levels of transporters, peptidases, receptors, G-coupled receptors, and ion channels (Yuan et al., 2019). Priming endometrial MSCs with interferon-γ (IFN-γ) increased immunomodulatory proteins in their Evs (Marinaro et al., 2019). Serum deprivation modified the composition of UC, AT, or BM-MSC-derived Evs for 24 h in order to improve the delivery of exosomes, but not their microvesicles, to target neurons (Haraszti et al., 2019). Hypoxia also changed the proteomic profile of Evs in MSCs, with an increase in proteins related to growth factors and a decrease in proteins related to oxidative metabolism (Gessner et al., 2021; Gregorius et al., 2021). A 3D culture using CellHesion^®^ VP increased EV production by MSCs and changed their protein profile, with an increase in proteins related to immune response (Kim E. S. et al., 2021). Priming of MSCs with toll-like receptor III agonist poly(I:C) upregulated EV proteins related to immune response, among other processes (Pierce and Kurata, 2021). Exosomes derived from human AT-MSCs stimulated by LPS increased in angiogenic potential (Wu et al., 2021). Preconditioning of MSCs with IFN-γ induced release of Evs containing annexin-1, lactotransferrin, and aminopeptidase N (Takeuchi et al., 2021). Priming of MSCs with melatonin increased ubiquitin-specific protease 29 in their Evs, which promoted increased microglia activation to the M2 phenotype and resulted in better recovery of motor behavior (Liu W. et al., 2021). Another molecule capable of enhancing the release of EVs and their therapeutic effect is metformin (Liao et al., 2021).

### miRNAs Related to MSC-Derived Evs

Many groups have shown that MSCs release miRNA-containing Evs that modulate molecular pathways in the target cell. miRNAs are non-coding RNAs approximately 22 nucleotides long, and are involved in post-transcriptional regulation of gene expression (Asgarpour et al., 2020). The importance of miRNA in the therapeutic effects of MSC-derived Evs is evidenced by inhibiting miRNA maturation or function. Knockdown of argonaute-2, a key protein for miRNA function, abolishes the protective effects of MSC-derived Evs in a model of optic nerve injury (Mead and Tomarev, 2017). The miRNA content of BM-MSC-derived Evsproved to be important for the neuroprotective effect in a rat model of chemobrain (El-Derany and Noureldein, 2021). Certain specific miRNAs were reported to have a pivotal role in the MSC-derived Ev effect. Endometrial MSCs primed with IFN-y 18 significantly altered miRNAs (Marinaro et al., 2019). Evs derived from hydrogen sulfide-primed BM-MSCs had an intensified therapeutic effect in a model of hypoxia-ischemia through miR-7b-5p activity (Chu et al., 2020). BM-MSCs transfer mir-29b-3p to neurons through Evs, preventing hypoxic-ischemic injury (Hou et al., 2020). Inhibition of mir-21-5p in BM-MSC-derived Evs attenuated the functional recovery of rats subjected to a spinal cord injury (Zhou et al., 2019). Overexpression of CDKN2B, a downstream target for mir-106b, reversed the effect of mir106b-containing MSC-derived Evs on a mouse model of Parkinson’s disease (Bai et al., 2021). Interestingly, the overexpression of miRNAs in MSCs may be reflected in their respective Evs. Mir-29b-3p is accumulated in Evs derived from BM-MSCs overexpressing this miRNA, and transfection with mir-29b-3p inhibitor downregulated it in respective Evs (Hou et al., 2020). Transfection of BM-MSCs with the mir-17-92 cluster enriched their Evs and improved the tissue and functional behavior of rats subjected to a brain-injury model (Zhang Y. et al., 2021). Silencing mir-29c-3p also reduced the effect of BM-MSC-derived Evs in an AD model (Sha et al., 2021). The miR-125a found in BM-MSCs promotes M2 macrophage polarization, which was attenuated by the knockdown of this miRNA (Chang et al., 2021). Transfection of AT-MSCs with miR-22 mimics resulted in a mir-22 enriched pool of Evs and reduced inflammation in a mouse model of AD (Zhai et al., 2021). Also, Evs of MSCs transfected with miR-26a-5p and miR-221-3p mimic, respectively, attenuated ischemia/reperfusion injury and stroke (Ai et al., 2021; Cheng et al., 2021). A gain- and loss-of-function study showed that mir-26a is important for the neuroprotective effect of Evs from AT-MSCs in a model of ischemia/reperfusion (Hou et al., 2021). A recent study showed that viral transduction of MSCs may impact their miRNA cargo in Evs (Zubkova et al., 2021). Priming MSCs with conditioned medium of activated microglia changed the miRNAs of Evs to a more immune-regulatory profile, which resulted in inhibition of microglial and astrocyte activation and better behavioral function (Markoutsa et al., 2022). Other stimuli can alter the miRNA content in MSC-derived Evs, such as exposure to glioblastoma-derived microvesicles (Garnier et al., 2022).

### Long Noncoding RNAs Related to MSC-Derived Evs

Other noncoding RNAs found in MSC-derived Evs are long noncoding RNAs (lncRNAs) and circular RNAs (circRNAs). To date, little is known about the roles of circRNAs and lncRNAs in the therapeutic effect of MSC-derived Evs, especially for neurodegenerative diseases. CircRNAs have been shown to regulate gene expression by interacting with RNA-binding proteins and functioning as miRNA sponges (Hansen et al., 2013; Zang et al., 2020). Recently, transfection of AT-MSCs with cicrAkap7 proved to enhance the neuroprotective effect of their Evs in a mouse model of ischemic injury (Xu et al., 2020). CircRNAs have also been implicated in the therapeutic effect of Evs in several models, by regulating angiogenesis (Zhang J. et al., 2021) and reducing pyroptose and cytotoxicity (Cao et al., 2020; Yan et al., 2020). LncRNAs are the most abundant class of non-coding RNAs, with >200 nucleotides (Policarpo et al., 2021). In MSC-derived Evs, the lncRNA SNHG7 showed protective potential in a model of diabetic retinopathy (Cao et al., 2021).

### *In vivo* Delivery of MSC-Derived Evs

Treatment with Evs can be performed by either local or systemic delivery, depending on the target and the injury model. Because of their very small size, Evs can be easily systemically injected without the risk of embolism. BM-MSC-derived Evs can accumulate in the infarcted hemisphere after injection into the tail vein in rats in a stroke model (Moon et al., 2019). Homing of Evs to injured organs may be favored by their expression of chemokine receptors such as CXCR4 (Kim S. J. et al., 2012). Furthermore, Evs can cross the blood-brain barrier, although this mechanism is not fully understood (Matsumoto et al., 2017). Other organs can uptake Evs, as human MSC-derived Evs were found in liver, spleen, and BM 6 h after intravenous injection (Wen et al., 2019). MSC-derived Evs injected intraocularly (Mead and Tomarev, 2017) or systemically (Seyedrazizadeh et al., 2020) can protect retinal ganglion cells. Recently it was shown that endometrial MSC-derived Evs were neuroprotective in a hippocampal injury model after intranasal administration (Leon-Moreno et al., 2020). Intranasally administered human BM-MSC-derived Evs can reach the intact or injured forebrain (Kodali et al., 2019). Systemic or intranasal administration of AT or BM-MSC-derived Evs protect neurons and decrease the memory deficit in mice with AD (Leon-Moreno et al., 2020; Losurdo et al., 2020). Furthermore, Evs from BM, as well as UC-MSCs inhibit apoptosis and improve functional recovery in rodents subjected to brain ischemia (Han et al., 2020; Seifali et al., 2020; Xin et al., 2020). BM-MSC-derived Evs systemically injected in rats subjected to subarachnoid hemorrhage protected neurons from apoptosis (Gao et al., 2020).

## MSC Modifications

### MSC Preconditioning

#### Pretreatment With Hypoxic Conditions

In order to enhance the therapeutic potential of MSCs, to improve their proliferative and survival rate, which is important considering the need for a large number of cells for transplants, or to improve the secretion of factors related to neuroprotection or reduction of inflammation, studies have evaluated the effects of pre-treatment of MSCs, using different approaches (Omid Sadatpoor et al., 2020).

In recent decades, studies have evaluated the effects of hypoxic conditions on the biological characteristics of MSCs. Oxygen concentration is an important factor in the function of stem cells (Simon and Keith, 2008). An atmosphere of 1%–5% O_2_ shows effects on MSCs compared to normoxic conditions (20%) in relation to proliferation, differentiation, migration capacity, and metabolism (Grayson et al., 2006; Potier et al., 2007; Rosová et al., 2008; Hung et al., 2012; Ejtehadifar et al., 2015; Kakudo et al., 2015; Elabd et al., 2018). In a model of intracerebral hemorrhage, olfactory mucosa MSCs under hypoxia alleviated cellular senescence, with a 1.2-fold decrease in the percentage of β-galactosidase-positive cells compared to cells under normoxia. Additionally, an enhancement of autophagy promoted by the increase in miR-326 expression could be seen in MSCs under hypoxia (Liu J. et al., 2021). Moreover, when cultured under 5% O_2_, significant increases in proliferation of MSCs at day 5 and in the expression levels of markers such as C-Myc and Nestin were observed. A reduction in the number of β-galactosidase-positive cells was observed in all MSCs under hypoxia compared to those in normoxia, demonstrating that hypoxic conditions affect not only the proliferative potential of MSCs but also the senescence process, which could interfere in the biological activity of the cells, thus preventing the desired effects after transplantation (Kwon et al., 2017). In another study, an atmosphere of 2% O_2_ affected the paracrine effects of aged BM-MSCs in an *in vitro* model of ischemic stroke. The conditioned medium of aged BM-MSCs under hypoxia showed an increase in VEGF levels from 2 to 5–3 to 6 ng/ml, demonstrating that the treatment was able to achieve the therapeutic level of above 1 ng/ml. Therefore, hypoxia treatment of MSCs seems to contribute to the neuroprotective effects of these cells after ischemic stroke. Interestingly, the cells used in the study were from patients more than 70 years old, which indicates that the treatment could also contribute to restoring the beneficial effects of cells derived from elderly donors since the age of the donors affects the biological characteristics of MSCs (Zhang et al., 2019a).

#### Pretreatment With Growth Factor and Cytokines

Preconditioning with growth factors, cytokines, and other biomolecules has been used to simulate the target microenvironment of MSCs after transplantation, in order to promote the prior adaptation of cells and secretion of important molecules (Hu and Li, 2018).

The immunomodulatory activity of MSCs is widely discussed as one of the most prominent roles of these cells. However, other than being beneficial to modulate the inflammation site, the secretion of growth factors and cytokines also has an autocrine effect on MSC behavior, interfering with their proliferation and differentiation (Eom et al., 2014). As a strategy to enhance those autocrine effects, it is possible to manipulate MSC behavior *in vitro* by adding certain growth factors and cytokines to the culture, increasing cell survival, migration, and cytokine secretion (Liu et al., 2011). Bone morphogenetic protein (BMP)-3, fibroblast growth factor (FGF), vascular endothelial growth factor (VEGF), and epidermal growth factor (EGF) are the best factors to pretreat the MSCs to increase cell proliferation and survival (Rodrigues et al., 2010). Other than optimizing culture conditions, growth factors and cytokines can be used to induce MSC differentiation toward neural identity *in vitro*; some studies have demonstrated transdifferentiation of MSCs into neural progenitor cells, using EGF and bFGF together with neural induction factors such as N-2 and B-27 (Peng et al., 2019).

Another study using an *in vitro* model of LPS-induced neuroinflammation managed to reduce the secretion of IL-6 and TNF-α by 35% and 41%, respectively, in addition to a 13-fold increase in the secretion of IL-10 by microglial cell line BV2 after incubation in conditioned medium of MSCs preconditioned with the cytokine IL-1. This anti-inflammatory effect was related to the increased secretion of G-CSF in the supernatant of the MSCs against the pro-inflammatory environment generated by preconditioning with IL-1 (Redondo-Castro et al., 2017). Likewise, in the model of periventricular leukomalacia induced in neonatal rats, treatment with human UC-MSCs preconditioned with IFN-γ increased the preservation of tissue myelin basic protein (MBP) by about 18%, while treatment with control MSCs reached only 2.5% protein preservation, both compared to the untreated group. Genetic analysis of cells after preconditioning revealed an increase in the expression of TGS-6 and IDO, proteins with anti-inflammatory functions, which may explain the results (Morioka et al., 2017).

In a more targeted method, MSCs conditioned with serum from stroke victims were more effective in promoting functional gains and reducing the Modified Neurological Severity Score (mNSS) of animals with induced ischemic stroke, compared with groups treated with cells stimulated by serum from healthy donors or FBS. Analysis of the gene expression of the treated cells showed an increase in the expression of 88 proteins in relation to the controls, including important factors related to cellular communication and signal transduction, such as FGF, VEGF, EGF, BNDF, and MMP-9 (Moon et al., 2018). Preconditioning of MSCs with TGF-β was able to increase homing of MSCs carrying a lethal gene to treat a model of glioblastoma in mice. Conditioning with TGF-β increased expression of the CXCR4 receptor in MSCs, resulting in an increase in the efficiency of these cells in delivering the therapeutic gene, and consequently a 50% reduction of the tumor volume in relation to the group treated with unstimulated MSCs (Li et al., 2019). In a model of multiple sclerosis in mice, treatment with preconditioned MSCs with SDF-1α also promoted an increase in CXCR4 expression in transplanted cells (about six times higher than the levels expressed by unconditioned MSCs), which led to a significant reduction in apoptosis of these cells, thus promoting greater myelination and improving the spatial learning and memory of treated animals compared to control groups (Beigi Boroujeni et al., 2020). Taken together, these data suggest that preconditioning with cytokines and growth factors is an effective method to promote increased expression of receptors and molecules of interest in MSCs, which can improve their therapeutic potential for different neurological diseases.

#### Pretreatment With Pharmacological Agents and Bioactive Molecules

Despite the recognized potential of MSCs to promote neuroprotection and tissue repair in CNS lesions and diseases *via* biomolecule secretion (Uccelli et al., 2011), some extrinsic factors, such as the source of achievement, donor characteristics, and culture conditions can interfere with their secretory activity (Hagmann et al., 2013; Elahi et al., 2016; Heathman et al., 2016). Therefore, *in vitro* preconditioning of MSCs with a variety of active substances, ranging from inorganic compounds to pharmacological agents, has been explored to increase or standardize the therapeutic potential of MSCs.

Preconditioning with pharmacological agents has been used to optimize the secretory profile of MSCs for application in different models. Linares et al. (2016) investigated the effect of preconditioning BM-MSCs with a combination of mood-stabilizing drugs, lithium chloride, and valproic acid, for the treatment of a Huntington’s disease model in rats. This preconditioning resulted in an increase in the expression of genes involved with trophic effects, stress response, antioxidants, and anti-apoptosis, among others, which contributed to an increase of approximately 60% in the survival of the treated cells after the graft, compared to unconditioned MSCs, and contributed to a better functional performance for the group treated with preconditioned cells. In another study, the conditioned medium of AT-MSCs preconditioned with the iron chelator deferoxamine (DFX) was effective in promoting neuroprotection and apoptosis reduction in a culture of DRG neurons challenged by hyperglycemic insult. The reduced rate of apoptosis (1.6% for preconditioned MSCs vs. 3.5% for control MSCs) was related to increased expression of genes involved in neuroprotection, such as GDNF, NGF, and NT3 (Oses et al., 2017). The functional performance of rats in a stroke model due to middle cerebral artery occlusion (MCAO) increased after treatment with BM-MSCs that were preconditioned with sodium hydrosulfide (NaHS). This result was accompanied by a reduction of approximately 13.5% of the area affected by the infarction after treatment with MSCs induced with NaHS compared to the untreated group, due to the increased expression and secretion of VEGF and BDNF in MSCs (Zhang et al., 2016).

#### MSCs in Three-Dimensional Cultures

An innovation of tissue engineering, a three-dimensional culture of MSCs such as spheroid culture or 3D culture using biomaterials such as scaffolds and hydrogels may confer improvements in MSC properties compared to two-dimensional culture methods.

3D MSC cultures show increased rates of cell proliferation, viability, and survival compared to 2D cultures (Baraniak and McDevitt, 2012; Alimperti et al., 2014; Murphy et al., 2014), in addition to maintaining their multilineage differentiation potential, favoring their use in cartilage and bone-repair models (Murphy et al., 2014; Occhetta et al., 2015; Yan and Wu, 2020). On the other hand, in the case of 3D MSC cultures with biomaterials, physical characteristics related to the surrounding environment, such as topography, surface tension, and matrix rigidity are able to define the degree of differentiation and the fate of MSCs, without the need for additional biochemical signals. For example, the use of soft, elastic, or rigid matrices can direct MSC differentiation into neural cells, myocytes, or osteoblasts, respectively (Wang et al., 2010; Wen et al., 2014).

The immunomodulatory properties of MSC are also regulated by 3D culture. Several studies using cultures of MSC spheroids derived from different sources demonstrated that this configuration was more effective in promoting the secretion of immunosuppressive factors such as PGE2, TSG-6, and STC-1 (Bartosh et al., 2013), in addition to decreasing the proliferation of immune cells (Miceli et al., 2019) and inducing macrophage polarization for an anti-inflammatory profile (Ylöstalo et al., 2012; Vallés et al., 2015). Similar results were obtained from 3D MSC cultures in hydrogels, with increased secretion of PGE2 and TSG-6, in addition to HGF (Papadimitropoulos et al., 2014; Follin et al., 2015).

MSC spheroids also showed advantages compared to two-dimensional culture, in the secretion of angiogenic factors such as HGF, PDGF, TGF-β, VEGF, FGF1, GRO-α, SDF-1, and EGF, and were able to induce increases in tube formation and capillary maturity (Park et al., 2014). The secretome of MSC grown in scaffolds was more efficient in promoting healing in *in vitro* models of corneal wounds, compared to the secretome obtained from a 2D culture. This result was related to higher levels of HGF and ICAM-1 present in the secretome of a 3D culture compared to the concentrations produced by the monolayer (Carter et al., 2019). The difficulty of gas exchange and the gradient of cytokines created within the 3D cultures are related to the increased expression of HIF-1 and HGF, explaining their greater regenerative potential (Bhang et al., 2011).

### Genetically Modified MSCs

As discussed above, many of the regenerative and protective effects evoked by MSC therapy in CNS are due to a paracrine action, mediated by trophic factors, cytokines, and other molecules secreted by these cells. Genetic engineering of MSCs to overexpress and/or secrete specific molecules of interest in the damaged tissue is a valuable tool to enhance their therapeutic potential (Nolta, 2016). An increase in the efficacy of MSC therapy also implies a reduction in the number of cells required per dose and consequently lowers the cost of large-scale cell production. Several virus-based approaches have been used for genetic engineering of MSCs, including retrovirus, lentivirus, and adenovirus, each of which has advantages and limitations. Recently, an efficient non-viral process using a cationic polymer for MSC programming has been described (Ho et al., 2020). Other approaches, such as plasmid transfection, can also be used to modify MSCs, but viral vectors are still the most widely used tool for genetic engineering of MSCs in preclinical trials, as in the majority of studies described in this section. Therefore, numerous MSC lines overexpressing several trophic factors have been developed and tested in models of neurological diseases in recent years (for a detailed review of engineered MSCs, different modification methods, and applications in regenerative medicine, see Damasceno et al., 2020). Neurotrophic factors, such as nerve growth factor (NGF), brain-derived neurotrophic factor (BDNF), and neurotrophin 3 (NT-3) and 4 (NT-4), are a family of soluble proteins involved in a plethora of functions but mainly related to neuronal survival. Mouse BM-MSCs overexpressing BDNF were tested in a model of chronic retinal degeneration. Intravitreal injection of BDNF-MSCs promoted a reduction of apoptotic markers in the retina, resulting in long-term neuroprotection of photoreceptors and better functional outcomes in electroretinography analysis. In all parameters, BDNF-MSCs induced better results than non-modified MSCs, suggesting that genetic engineering improved the therapeutic efficacy of the MSCs (Lejkowska et al., 2019). Intravitreal injection of erythropoietin-expressing MSCs also increased photoreceptor neuroprotection in a model of sodium iodate-induced retinal degeneration, as shown by electroretinography (Koh et al., 2021). Neuroinflammation is another key aspect of several neurodegenerative conditions. Intracerebral injection of BM-MSCs overexpressing interleukin IL-13 in cuprizone-induced multiple-sclerosis mice induces anti-inflammatory microglial activation and reduces demyelination and oligodendrocyte loss, effects not achieved with naïve MSC therapy (Le Blon et al., 2013).

An important aspect regarding the efficacy of cell therapy is that the MSC secretome can be directly modulated by the often-unfavorable host environment (Wang et al., 2014). Therefore, genetic modification can also be used to improve MSC survival, homing, and grafting after transplantation. In a mouse model of acute spinal cord injury, BM-MSCs overexpressing human insulin-like growth factor 1 (hIGF1-MSCs) have increased survival after transplantation in relation to naïve MSCs. Therapy with hIGF1-MSCs reduced demyelination and promoted more-robust functional recovery than naïve MSCs (Allahdadi et al., 2019). Human MSCs overexpressing fibroblast growth factor (FGF) by plasmid transfection also induced motor improvement in a spinal cord-injury model; the proposed mechanism of this effect was through increased differentiation of endogenous neural stem cells (Huang F. et al., 2021). Although systemic circulation and interaction with other cell types can be important for the activation of MSCs, an increase in the number of cells that reach and remain in the injury site can result in optimized therapeutic effects. Several reports in murine models have indicated that a few hours after systemic transplantation, MSCs tend to accumulate in the lungs, and very few cells reach the brain or the spinal cord (Dos Santos Ramalho et al., 2019; Mello et al., 2020). MSCs overexpressing FGF increased their ability to migrate toward the lesion site after intracerebroventricular injection in a mouse model of traumatic brain injury, although the study did not assess differences in functional outcomes (Shahror et al., 2019). Although overexpression of integrin α4, a protein related to transendothelial migration, did not increase homing of rat MSCs in a model of stroke, it reduced cell aggregation and cerebral embolism, increasing the safety of therapy (Cui et al., 2017). Human UC-MSCs overexpressing the chemokine CCL2 by plasmid transfection showed increased migration to the injury site in rats submitted to middle cerebral arterial occlusion, a classic model of stroke. Moreover, in the acute phase of lesion, CCL2-MSC-treated animals showed a significant reduction in lesion volume and improved functional outcome in comparison to subjects treated with non-modified MSCs (Lee et al., 2020). Similarly, a previous study using the same model showed that MSCs overexpressing CCR2, the CCL2 receptor, had increased migration rates to the injury site and induced improvement in behavioral tests, due to the preservation of the blood-brain barrier integrity (Huang et al., 2018). Huang Y. et al. (2021) recently showed that small interfering RNA-mediated ablation of CUEDC2, a novel protein related to cancer and oxidative stress, in rat MSCs increased the efficacy of cell therapy in reducing the infarcted area after stroke induction. Finally, human MSCs that have been genetically modified are already being tested in clinical trials, and some of these studies are discussed in this review.

## MSC Therapy in Neurological Diseases

In the following sections, we briefly discuss the most recent studies regarding the safety, efficacy, and mechanism of action of MSC-based therapy in several neurological conditions, including amyotrophic lateral sclerosis, glaucoma, stroke, spinal cord injury, and autism. Most of the preclinical data described here were obtained from tests on animals, usually rats and mice, submitted to allogeneic or xenogeneic transplantation of human MSCs. Lesioned and transgenic rodent models have been essential not only for understanding the etiology and pathophysiology of these diseases, but also for evaluating the efficacy of different therapeutic approaches regarding MSC source, dosage, delivery route, and timing, as well as the possible adverse effects, providing remarkable insight into clinical translation of MSC transplantation to human patients.

### Amyotrophic Lateral Sclerosis

Amyotrophic lateral sclerosis (ALS) is a neurodegenerative disease that mainly affects the motor neurons (MN), leading to progressive muscle atrophy, paralysis, and death, usually 3–5 years after onset. The incidence of ALS is relatively constant worldwide, with a rate of 1–2 cases per 100,000 inhabitants (Brown and Al-Chalabi, 2017). Most ALS cases are idiopathic, termed sporadic ALS. Approximately 5%–10% of ALS cases are familial. Variations have been observed in approximately 120 genes that are associated with a risk of ALS, such as C9ORF72, SOD1, TARDBP, FUS, and VAPB, among others (Brown and Al-Chalabi, 2017). The discovery of genes associated with familial ALS allowed the development of animal models to study ALS (Philips and Rothstein, 2015), and more recently, the generation of patient-induced pluripotent cells has also contributed to understanding the diversity and complexity of ALS (Vasques et al., 2020).

It is not known exactly what process triggers the degeneration of MN. ALS is considered a multifactorial disease, where different mechanisms seem to be involved in its development. Some of the proposed hypotheses include protein aggregation, glutamatergic excitotoxicity, glial cell toxicity, neuroinflammation, oxidative stress, mitochondrial dysfunction, lack of growth factors, and dysfunction in axonal transport, among others (Mejzini et al., 2019).

Currently, there are no effective treatments for ALS. Many groups have been testing cell therapy as an alternative, especially with MSCs. Different routes of administration, doses, and injection times have been tested. BM is the main source of MSCs tested in animal models of ALS. These cells, derived from humans or rodents, have shown promising results, delaying disease progression and increasing animal survival (Zhao et al., 2007; Zhang et al., 2009; Kim et al., 2010; Forostyak et al., 2011, 2014; Uccelli et al., 2012; Zhou et al., 2013; Řehořová et al., 2019). As an alternative, some groups have tested BM mononuclear cells (BMMCs), a heterogeneous population of cells that contain a small percentage of both MSCs and hematopoietic stem cells. Preclinical studies using BMMCs have shown modest positive results; in some cases, the treatment delayed disease progression but with no change in survival (Gubert et al., 2016, 2019; Martínez-Muriana et al., 2020). Other sources of MSCs, such as AT and UC, have also been tested in ALS models (Marconi et al., 2013; Kook et al., 2020; Ciervo et al., 2021). Both showed some positive results, although more studies using these sources are important to compare the efficacy of these sources.

Although most preclinical studies using MSCs for ALS have shown positive results, this appears to vary according to the dose and injection site. Habisch and co-workers (2007) showed that 10^5^ human BM-MSCs injected intrathecally in SOD1-G93A mice had no effect on animal survival and delayed disease progression only in females (Habisch et al., 2007). Zhou and co-workers, using the same animal lineage, showed that a single intrathecal injection with a dose 5× higher of human BM-MSCs had a modest effect on motor performance and survival, but multiple injections resulted in a positive outcome (Zhang et al., 2009; Zhou et al., 2013). Testing different doses (10^4^, 2 × 10^5^, or 10^6^), Kim et al. (2010) showed that only the higher doses produced a positive outcome, especially 10^6^ human BM-MSCs. After intrathecal injections, few or no cells were found in the regions most affected by the disease (Zhang et al., 2009; Forostyak et al., 2014). Despite that, using multiple injections, intrathecal cell therapy modulates neuroinflammation, as observed by the reduction of microgliosis and astrogliosis as well as decreased expression of iNOS and TNF-α (Zhou et al., 2013).

As an alternative to the intrathecal route, some groups have tested intraventricular injections. However, this route has not shown promising results. Transplantation of 2.5 × 10^5^ UC-MSCs protected MN and decreased neuroinflammation but had no effect on disease progression and animal survival (Sironi et al., 2017). In addition, lateral ventricle injection of human BM-MSCs had a negative effect on both motor performance and survival (Bursch et al., 2019). The authors attributed these results to an increase in microgliosis.

In order to directly access the site of MN degeneration, some groups have tested intraspinal injections. Vercelli and co-workers showed that 10^5^ human BM-MSCs injected into the lumbar spinal cord slowed disease progression (Vercelli et al., 2008). Using the same route, our group showed that BMMCs could also affect motor performance, but only when the cells were administered in the pre-symptomatic phase (Gubert et al., 2016). In agreement with these results, Bursch and coworkers showed an effect on disease progression but with no change in animal survival after repeated intraspinal injections of human BM-MSCs (Bursch et al., 2019).

In ALS, muscle atrophy results from the loss of neuromuscular junctions (NMJ). Considering that it is necessary not only to protect the motor neurons but to keep them functional, some groups have tested intramuscular injections of MSCs. Intramuscular injections of MSCs overexpressing GDNF for three consecutive weeks preserved NMJ, slowed disease progression, and increased animal survival (Suzuki et al., 2008). Recently, Kook et al. (2020) showed that repeated intramuscular injections (gastrocnemius muscle) performed once a week from the 12th week of life also had positive effects on motor performance and survival. They attributed these results to modulation of the iNOS pathway and a reduction in levels of intracellular reactive oxygen species (ROS) in the muscle (Kook et al., 2020).

In ALS, the immune system has been increasingly studied. Many research groups have tested intravenous injection as an alternative route. In addition to being a less invasive and easy to administer route, studies using this approach have shown positive results, such as a reduction in disease progression and an increase in animal survival (Zhang et al., 2007; Uccelli et al., 2012). Interestingly, these effects were observed even when the cells were administered after the onset of symptoms. As mechanisms, the authors observed a reduction in the numbers of microglia and astrocytes, as well as a reduction of IL-β and TNF-α expression (Uccelli et al., 2012).

Each of the routes mentioned above could affect different components of ALS. Therefore, many groups have proposed combining routes of administration to achieve a better outcome. Forostyak and co-workers reported positive effects on disease progression and survival after intravenous and intraspinal administrations at the onset of the disease (Forostyak et al., 2011). The outcome improved when intrathecal and intramuscular injections were combined, rather than when using the routes individually (Řehořová et al., 2019). The injections were administered three times, at onset and after 14 and 28 days. The authors reported neuroprotection, modulation in necroptosis pathways, and reduction of NF-κB and TNF-α expression.

Clinical trials with MSCs in ALS patients are underway. Most of the groups have used autologous MSCs and demonstrated that these cells are safe (detailed information regarding MSC-based clinical trials in ALS in recent years is summarized in Table 1). A meta-analysis study analyzing data for 152 patients treated with MSCs indicated a transitory effect of these cells after intrathecal administration (Morata-Tarifa et al., 2021). One study showed that patients with ALS who received intrathecal autologous transplants could be divided into two groups: responsive and non-responsive to treatment. In the responsive group, MSCs had higher VEGF expression than MSCs in the non-responsive group (Kim et al., 2014). This demonstrates that there are differences between MSCs, justifying the search for a source that can generate more-homogeneous cells with a higher capacity to express trophic factors and cytokines. In line with this idea, a randomized placebo-controlled phase 3 test was performed with about 189 patients. Autologous BM-MSCs modified to secrete higher levels of neurotrophic factors (NurOwn^®^) were injected intrathecally three times (at 8-week intervals). The therapy was safe and, although it did not reach the designated primary end-point, analysis of a subgroup of patients that were at a less severe stage of the disease revealed a significant preservation of function compared to the placebo (Cudkowicz et al., 2022). Therefore, MSC therapy for ALS seems promising, but considering the enormous heterogeneity of the patients, larger clinical trials are needed.

**Table 1:** Clinical trials using mesenchymal stem/stromal cells in amyotrophic lateral sclerosis.

Identifier and reference	Recruitment status	MSC source	MSC dose	Delivery route	Main results
**NCT02987413**	Completed	Not available (autologous)	1 × 10^8^ cells (2×)	Intrathecal	No results posted
**NCT03268603**	Recruiting	Adipose tissue (autologous)	1 × 10^8^ cells (every 3 months; total of 4)	Intrathecal	No results posted
**NCT01609283**	Completed	Adipose tissue (autologous)	1 × 10^7^ cells (1×) 5 × 10^7^ cells (1×) 5 × 10^7^ cells (2×) 1 × 10^8^ cells (1×) 1 × 10^8^ cells (2×)	Intrathecal	No results posted
**NCT01142856**	Completed	Adipose tissue (autologous)	1 × 10^6^ cells	Intrathecal	No results posted
**NCT02881489**	Unknown	Bone marrow (autologous)	Not available	Intrathecal	No results posted
**NCT01777646** (Petrou et al., 2016)	Completed	Bone marrow secreting neurotrophic factors (autologous)	94 × 10^6^ cells 141 × 10^6^ cells 188 × 10^6^ cells	Intrathecal and intramuscular	Therapy was safe and well-tolerated.
**NCT02492516**	Completed	Adipose tissue (allogeneic)	2 × 10^6^ cells/kg	Intravenous	No results posted
**NCT02881476** (Barczewska et al., 2019)	Unknown	Wharton’s jelly	0.42 × 10^6^ cells/kg	Intrathecal	Therapy was safe and well tolerated
**NCT01494480**	Unknown	Umbilical cord	Not available	Intrathecal	No results posted
**NCT03828123** (Sykova et al., 2017)	Completed	Bone marrow (autologous)	15 ± 4.5 × 10^6^ cells	Intrathecal	Therapy was safe and well-tolerated. Reduction in ALSFRS decline.
**NCT01759797** (Nabavi et al., 2019)	Completed	Bone marrow (autologous)	2 × 10^6^ cells/ kg	Intravenous	Therapy was safe and well-tolerated.
**NCT01771640** (Nabavi et al., 2019)	Completed	Bone marrow (autologous)	2 × 10^6^ cells/ kg	Intrathecal	Therapy was safe and well-tolerated.
**NCT01051882** (Petrou et al., 2016)	Completed	Bone marrow secreting neurotrophic factors (autologous)	1 × 10^6^/kg cells (IT) 1 × 10^6^ cells/site (24 sites—IM)	Intrathecal or intramuscular	Therapy was safe and well-tolerated
**NCT02917681**	Unknown	Bone marrow (autologous)	Not available	Intrathecal	No results posted
**NCT02290886**	Completed	Adipose tissue (autologous)	3 × 10^6^ cells/kg 6 × 10^6^ cells/kg 12 × 10^6^ cells/kg	Intravenous	No results posted
**NCT03280056** (Cudkowicz et al., 2022)	Completed	Bone marrow secreting neurotrophic factors- MSC-NTF, NurOwn^TM^ (autologous)	Not available	Intrathecal	Therapy was safe and well-tolerated. Phase 3 study, only a subgroup of treated patients retained more function than placebo group.
**NCT02017912**	Completed	Bone marrow secreting neurotrophic factors- MSC-NTF, NurOwn^TM^ (autologous)	Not available	Intrathecal and intramuscular	No results posted
**NCT03296501** (Kuzma-Kozakiewicz et al., 2018)	Active, not recruiting	Adipose tissue (autologous)	1.6 × 10^4^ cells 5.6 × 10^7^ cells	Intraspinal and Intrathecal	Therapy was safe and well-tolerated.
**NCT04651855**	Recruiting	Wharton’s jelly	Not available	Intrathecal	No results posted
**NCT04745299**	Recruiting	Bone marrow (autologous) Lenzumestrocel (Neuronata-R^®^ Inj.)	Not available	Intrathecal	No results posted
**NCT04821479** (Petrou et al., 2021a)	Completed	Bone marrow (autologous)	1 × 10^6^ cells/kg (1–4 injections)	Intrathecal	Therapy was safe and well-tolerated. Signs of clinical efficacy, related to the intervals between administrations.
**NCT05003921**	Recruiting	Umbilical cord	5 × 10^7^ cells (three injections)	Intrathecal	No results posted

### Glaucoma and Retinal Lesions

Vision impairments affect the economy and quality of life and even increase the risk of death. It is estimated that 2.2 billion people worldwide have some visual impairment, and approximately 36 million of them are blind (Bourne et al., 2017; Williams et al., 2020). Cataract is the most common cause of blindness but is reversible in most cases (Williams et al., 2020). The leading cause of irreversible blindness, glaucoma, affects about 64 million people worldwide, of whom 6.9 million have some vision impairment or are blind (Williams et al., 2020). Glaucoma is characterized by degeneration of the optic nerve and death of retinal ganglion cells (RGCs), leading to progressive loss of vision. RGCs die mostly by apoptosis and, like other neurons, cannot be replaced. Furthermore, until now no clinical treatment exists to sustain RGC survival and promote the regeneration of axons to their central targets. Cell therapy is an alternative approach that has yielded promising results. BM cells were shown to provide long-term protection and RGC regeneration and synaptic reconnection in the superior colliculus in an optic-nerve crush model (Zaverucha-do-Valle et al., 2011, 2014; Mesentier-Louro et al., 2014, 2019). These were the first studies to report RGC reconnection resulting from cell therapy. Moreover, BM-MSCs were also shown to be neuroprotective in models of ocular hypertension (Emre et al., 2015; Mead et al., 2016). Although UC-MSCs were reported to have neuroprotective effects on RGCs subjected to an optic-nerve injury (Nascimento-Dos-Santos et al., 2019; Wang Y. et al., 2021), the effect was transient. In contrast, da Silva-Junior et al. (2021) recently reported that human WJ-MSCs could protect RGCs for a relatively long period and promote axonal regeneration to brain targets after intravitreal injection in an optic-nerve crush model. Millán-Rivero and colleagues (2018) showed that human UC-derived-MSCs protected RGCs and some integrated into the retina (Millán-Rivero et al., 2018). The same was reported with BM-MSCs (Li et al., 2009). These findings contrast with other reports that MSCs injected intravitreally did not integrate into the rat retina (Hill et al., 2009), and even after 18 weeks post-injection, the majority of BM-MSCs remained in the vitreous body (Mesentier-Louro et al., 2014). Importantly, a recent study indicated that care with the type of transplantation of cells (syngeneic, allogeneic, or xenogeneic) may impact the results, since MSCs from different donor species can exert different effects (Norte-Munoz et al., 2021).

Diabetic retinopathy is a complication of diabetes that results in damage to retinal blood vessels, and is expected to affect about one-third of diabetics by 2045 (Williams et al., 2020). To date, diabetic retinopathy is treated by laser coagulation, anti-VEGF, or glucocorticoid drugs; however, these methods have serious side effects and do not ensure the preservation of vision (Gaddam et al., 2019). Systemic treatment with BM-MSCs proved to be safe in patients with diabetic retinopathy (Gu et al., 2018). Intravitreally injected BM-MSCs integrated into the inner retina and improved the electrophysiological retinal function of the retina in rats in a model of diabetic retinopathy (Çerman et al., 2016). Systemic or local injection of UC-MSCs attenuated angiogenesis and inflammation in the retina in a model of diabetic retinopathy (Yu et al., 2020; Zhao et al., 2020). Overexpression of LIF in MSCs was shown to increase the neuroprotective effect in diabetic retinopathy (Chen et al., 2021).

Optic neuritis is an inflammatory demyelinating disorder of the optic nerve, often related to multiple sclerosis (Redler and Levy, 2020). Intraperitoneal injection of human BM-MSCs decreased RGC death and improved their function in a mouse model of multiple sclerosis (Gramlich et al., 2020). BM-MSCs administered intravenously proved to be safe for patients with neuromyelitis spectrum disorder and improved neurological disability, with recovery in neural structures in the optic nerve and retina for up 2 years after treatment (Fu et al., 2016). Another study reported positive effects after follow up for 18 months of treatment with UC-MSCs in patients with neuromyelitis optica (Lu et al., 2012).

Many clinical trials with MSCs for optical disease are registered on ClinicalTrials.org (Table 2). Studies using MSCs have reported safety and effectiveness in the treatment of patients with optic-nerve injuries (Kahraman and Oner, 2020; Sung et al., 2020). One study investigated 29 patients with optic atrophy, who were followed up to 1 year after suprachoroid UC-MSC implantation and showed improvement in visual parameters (Kahraman and Oner, 2020). Intravitreal injection of BM-MSCs in patients with retinitis pigmentosa resulted in visual improvement (Weiss and Levy, 2018; Tuekprakhon et al., 2021). Nevertheless, there is much concern about the clinical use of MSCs, since some reports described visual impairment in patients after intraocular injection of BM-MSCs (Kasetty et al., 2020). However, MSC-derived Evs are an alternative to avoid the presence of cells in the vitreous body. Mathew et al. (2021) reported the distribution of Evs on the retina *in vitro* and *in vivo*. MSC-derived Evs were rapidly cleared from the vitreous body and endocytosed by astrocytes, microglia, and neurons in a dose-dependent manner (Mathew et al., 2021). Evs from embryonic MSCs were shown to protect RGCs for at least 60 days and promote regeneration in an optic-nerve crush model (Seyedrazizadeh et al., 2020). MSC-derived Evs were shown to be neuroprotective in *in vitro* models of diabetic retinopathy (Beltramo et al., 2014). Evs were also reported to protect RGCs in a model of optic-nerve injury (Mead et al., 2016; Pan et al., 2019). In contrast, the angiogenic potential of MSC-derived Evs was reported to induce diabetes-like features of diabetic retinopathy *in vitro* (Beltramo et al., 2014). However, Evs have been shown to have protective effects on the retina in models of diabetic retinopathy (Fu et al., 2021; Li W. et al., 2021; Xu et al., 2021). Therefore, it is necessary to investigate which Ev components are undesirable for this treatment and if it is possible to direct MSCs to deliver Evs with appropriate molecules to enhance their effect.

**Table 2:** Clinical trials using mesenchymal stem/stromal cells in retinal diseases.

Identifier and reference	Recruitment status	MSC source	MSC dose	Delivery route	Main results
**NCT02638714**	Recruiting	Bone marrow	Not available	Not available	No results posted
**NCT01364246**	Unknown	Umbilical cord	Not available	Not available	No results posted
**NCT02249676** (Fu et al., 2016)	Completed	Bone marrow	1 × 10^8^ cells	Intravenous	MSC infusion is safe, reduces the relapse frequency, and mitigates neurological disability
**NCT01834079**	Unknown	Bone marrow	1 × 10^8^ cells	Intrathecal	No results posted
**NCT01920867** (Weiss et al., 2015a, b; Weiss et al., 2016a, b)	Unknown	Bone marrow	~3.48 × 10^8^ cells	Retrobulbar Subtenon Intravenous Intraocular Intra-optic nerve	Treatment with BMSCs is safe and provided improvement in vision.
**NCT03011541** (Weiss et al., 2017; Weiss and Levy, 2018, 2019; Weiss and Levy, 2020, 2021)	Recruiting	Bone marrow	~3.48 × 10^8^ cells	Retrobulbar Subtenon Intravenous Subretinal Intraocular Intravitreal	Treatment with BMSCs is safe and provided improvement in vision.
**NCT02795052** (Bhasin et al., 2012; Anbari et al., 2014; Weiss and Levy, 2016)	Recruiting	Bone marrow	54.6 × 10^6^ cells	Intravenous Intranasal	Cell therapy is safe and feasible, which may facilitate restoration of function in chronic ischemic stroke.
**NCT02330978**	Completed	Bone marrow	1 × 10^6^ cells	Intravitreal	No electroretinographic function. Retinal detachment with proliferative vitreoretinopathy was noted in one patient.
**NCT05147701**	Recruiting	Umbilical cord	1 × 10^6^ cells	Intravenous Subtenon	No results posted
**NCT04224207** (Özmert and Arslan, 2020a)	Completed	Wharton’s Jelly	2–6 × 10^6^cells	Subtenon	WJMSC treatment was safe and resulted in electroretinography
**NCT04315025**	Completed	Umbilical cord	Not available	Peribulbar	No results posted
**NCT01531348** (Tuekprakhon et al., 2021)	Completed	Bone marrow	1 × 10^6^ cells	Intravitreal	Intravitreal injection of BM-MSCs appears to be safe and potentially effective
**NCT04763369** (Kahraman and Oner, 2020; Özmert and Arslan, 2020a)	Recruiting	Umbilical cord	5 × 10^6^ cells	Subtenon Suprachoroidal	Suprachoroidal administration ofUC-MSCs has beneficial effect on visual function
**NCT00395200** (Connick et al., 2011, 2012)	Completed	Bone marrow	2 × 10^6^ cells/kg	Intravenous	Autologous mesenchymal stem cells were safely given to patients with secondary progressive multiple sclerosis. The evidence of structural, functional, and physiological improvement after treatment in some visual endpoints is suggestive of neuroprotection.
**NCT02144103**	Unknown	Adipose tissue	Not available	Subtenon	No results posted
**NCT03437759**	Active, not recruiting	MSC-derived exosome	20 or 50 μg	Intravitreal	No results posted
**NCT04877067** (Özmert and Arslan, 2020a, b)	Completed	Wharton’s Jelly	2–6 × 10^6^cells	Subtenon	No serious adverse events or ophthalmic/systemic side effects for 6 months follow-up
**NCT03173638**	Active, not recruiting	Bone marrow	Not available	Intravitreal	No results posted

### Stroke

Stroke is the second leading cause of death and the main cause of long-term disability, significantly reducing the patient’s quality of life and representing an important economic burden. Ischemic stroke is characterized by an interruption in blood flow to the brain, generally due to a blood clot, causing oxygen and glucose deprivation. The affected brain tissue can be divided into the core area, where neural death occurs by necrosis, and the penumbra area, in which neural cells are metabolically impaired but still able to recover if the blood flow is rapidly restored. Ischemic strokes are the most prevalent, accounting for 85% of cases, and the mortality rate is around 15% (Ganesh et al., 2016). The current FDA-approved treatment is the thrombolytic drug tissue plasminogen activator if administered within 4.5 h after onset of symptoms. After this time window, surgical removal of the clot is the remaining option. In hemorrhagic stroke, a vessel rupture allows extravasation of blood to the brain parenchyma, subarachnoid or intraventricular spaces. This bleed causes a primary lesion, inducing death of neural cells due to oxygen and glucose deprivation and mechanical pressure from blood. Blood metabolites trigger a secondary inflammatory response, contributing to an increase in the initial injury (Zheng et al., 2016). Although less frequent, the hemorrhagic-stroke mortality rate is around 50%, comprising 60% of stroke-related deaths (Yang et al., 2017). To date, the treatment options available for hemorrhagic stroke are limited to cranial decompression surgery, indicated in only a few cases; blood-pressure control; and life-support measures (Fogarty Mack, 2014).

In recent years, several preclinical and clinical studies have evaluated the therapeutic potential of MSC therapy in ischemic and hemorrhagic stroke (for reviews fully dedicated to MSC therapy in stroke, see Turnbull et al., 2019 and Li J. et al., 2021). In a model of focal ischemic stroke induced by thermocoagulation of blood vessels, our group demonstrated that in the acute phase of the insult, only one-tenth of the dose of BM-MSCs is needed to induce the same sensory-motor recovery as bone marrow-derived mononuclear cell therapy (de Vasconcelos dos Santos et al., 2010). A commonly used animal model of ischemic stroke is the middle cerebral artery occlusion (MCAO). Rats submitted to MCAO and treated with 3 × 10^6^ BM-MSCs injected systemically showed better results in neurological severity scores (NSS) 72 h after surgery, and lesion volume was also reduced in relation to the vehicle group. Interestingly, Nogo-A and its receptor, NgR, were downregulated in the ischemic core in the BM-MSC-treated group. Nogo-A inhibits central axon growth after lesion and inhibits neuronal repair after ischemia (Zhang J. et al., 2020). Intra-arterial injection of 5 × 10^5^ rat BM-MSCs 24 h after MCAO also induced a reduction in the infarct area and improvement of motor function. BM-MSC-derived mitochondria were detected in host cells post-transplantation, which was correlated with an increase in angiogenesis in the peri-infarcted area (Liu et al., 2019). Using the MCAO model, a recent report demonstrated that rat BM-MSCs pretreated with IFN-γ (aMSCγ) injected intravenously in the acute phase of the lesion-induced a better outcome than naïve MSCs. Although both aMSCγ- and naïve MSC-treated groups displayed similar results regarding motor function improvement, NSS, lesion volume reduction, and microgliosis attenuation in relation to the vehicle group, aMSCγ therapy also induced the proliferation of neural progenitor cells and oligodendrogenesis in the ipsilateral subventricular zone (Tobin et al., 2020). BM-MSCs expressing the BDNF receptor TrkB (BM-MSC-TrkB) directly injected into the penumbra area 5 days after MCAO, in combination with electroacupuncture therapy, improved motor function in the rotarod test 15 days after the lesion. BM-MSC-TrkB had higher survival rates post-transplantation and increased migratory ability to the lesion site in relation to naïve MSCs (Ahn et al., 2019). BM-MSC transplantation also improved neurological outcomes and reduced the infarcted area after MCAO in spontaneously hypertensive rats (Liu et al., 2022). It has recently been proposed that the beneficial effects of BM-MSC therapy in rats submitted to MCAO could be related to the regulation of the gut microbiome (Zhao et al., 2021). In a chronic model of ischemic stroke, 8 weeks after MCAO induction, three weekly injections of MSCs promoted a better functional recovery than a single injection, an effect that seems to be correlated to increased preservation of corpus callosum fibers (Takemura et al., 2021).

Human-derived MSCs also exert important neuroprotective effects in rats submitted to MCAO. Stereotaxic injection of 1 × 10^7^ human BM-MSCs into the cerebral cortex 3 days after injury-induced functional preservation in relation to the vehicle, up to 4 weeks post-lesion. Therapy also decreased the infarcted area and increased the expression of neural markers, such as βIII-Tubulin and GFAP (Xie et al., 2019). Combined therapy of human BM-MSCs with minocycline, a broad-spectrum antibiotic that inhibits microglial activation, resulted in better effects than isolated therapies. The group that received both therapies— 1.5 × 10^6^ cells injected intravenously 72 h after MCAO and 5 g/ml minocycline injected intraperitoneally daily for 2 weeks post-surgery—had improved NSS results and better motor outcome 21 days after the lesion, as well as a reduced infarcted area (Cho and Jeun, 2018). Culture methods also can improve the efficacy of MSC therapy. Three-dimensional culture of human BM-MSCs increased the persistence of injected cells around the injury site and improved the functional outcome in rats submitted to MCAO in relation to a standard monolayer culture (Yuan et al., 2019). Donor age is another important determinant of the regenerative capacity of human MSCs. Human BM-MSCs from older donors secrete less BDNF *in vitro* than younger MSCs. Rats submitted to MCAO treated with younger human BM-MSCs showed better functional recovery than the group treated with older human BM-MSCs (Yamaguchi et al., 2018).

Our group has recently demonstrated that intravenous injection of 3 × 10^6^ human WJ-MSCs reduced the hematoma volume in rats submitted to moderate intracerebral hemorrhage (ICH) by intrastriatal collagenase injection. Animals treated with human WJ-MSCs showed no significant functional difference in relation to the sham group in the elevated body swing test 8 days after lesion, differently from vehicle-treated animals, which displayed an increased percentage of swings to the contralateral side of the lesion (Mello et al., 2020). Human placenta-derived MSC therapy also reduced mortality from 50% to 8% in relation to the vehicle, when injected *via* tail vein in the acute phase of moderate collagenase-induced ICH. Hematoma volume was also significantly reduced in animals that received cell therapy, as well as brain edema, ventricular enlargement, and neuronal death (Choi et al., 2018). Similarly, rat AT-MSCs injected directly into the peri-lesioned area 48 h after collagenase injection in mice reduced the brain edema, improving the functional outcome. These positive effects were attributed to a reduction in apoptosis in the hematoma area and diminished expression of pro-inflammatory markers and aquaporin-4, a water channel found in astrocytes also associated with inflammation and edema formation (Zhang et al., 2019b). Intraventricular injection of BM-MSCs exerted similar neuroprotective effects in a rat model of ICH due to autologous blood injection: functional improvement along with reduced levels of IL-1α, IL-6, and IFN-γ (Huang et al., 2019).

MSC-derived products have already been tested in stroke models. Intranasal injection of human AT-MSC-derived Evs 24 h after a thermocoagulation ischemic lesion-induced a reduction in infarct volume and re-stabilization of vascularization in the peri-infarct area, culminating in behavioral improvement (Rohden et al., 2021). Recently, Zhao et al. (2020) showed that exosomes isolated from rat BM-MSCs overexpressing microRNA-223-3p injected into the tail vein 24 h and 14 days after MCAO induced an improvement in NSS, in relation to naïve-BM-MSC exosomes, but both groups were significantly different from the vehicle. The infarct area was also reduced in all exosome-treated groups, especially in microRNA-223-3p subjects. Interestingly, exosomes from rat BM-MSCs overexpressing microRNA-133b, related to functional recovery in spinal cord injury and cerebral ischemia, reduced apoptosis and the number of degenerating neurons in a rat model of hemorrhagic stroke (Shen et al., 2018). A previous study also indicated that knocking-down microRNA-133b in MSCs and their exosomes reduced the therapeutic potential of cell therapy after MCAO. Alternatively, MSCs overexpressing microRNA-133b induced a significant increase in functional recovery in relation to naïve MSCs, emphasizing the importance of this specific molecule in the beneficial effects of MSC therapy in ischemic stroke (Xin et al., 2013).

Several clinical trials using human MSCs in stroke have been conducted in recent years. In February 2022, 31 clinical trials using MSCs from different sources in stroke were registered at clinicaltrials.gov, most of them using ischemic patients. Several trials have shown that different doses of MSCs, injected through different delivery routes, are safe, and some have indicated a certain degree of efficacy (detailed information regarding active MSC-based clinical trials in stroke in recent years is summarized in Table 3). A phase I/II trial demonstrated the safety of intravenous transplantation of allogeneic BM-MSCs from a single donor, cultured under low oxygen (5%) conditions (ischemia-tolerant MSCs). The dose of 1.5 million cells/kg was used in 21 patients in phase II after it was considered safe in the prior phase in five individuals. The behavioral end points suggest significant neurological improvement after 12 months of follow-up (Levy et al., 2019). Different doses of allogeneic BM-MSCs genetically modified to express the Notch-1 receptor intracellular domain injected directly into the residual lesion area in chronic ischemic-stroke patients were also considered safe, although about 20% of the subjects showed adverse effects, more likely due to the surgical procedure than to the cell therapy itself. An improvement in clinical outcome after 12 and 24 months of follow-up, which correlated with a reduced lesion volume, was also suggested (Steinberg et al., 2016, 2018). Autologous BM-MSCs have been tested in a cohort of 16 subacute ischemic-stroke patients, where different cell doses, ranging from 10 × 10^7^–30 × 10^7^ and injected intravenously, were shown to be safe. An indication of functional improvement was detected (Jaillard et al., 2020). A recent *post hoc* analysis suggests that younger patient age and reduced time after the ischemic injury to start the therapy were the main factors related to lower limb recovery after autologous MSC transplantation in ischemic stroke patients (Chang et al., 2021). There is currently only one active trial using hemorrhagic-stroke patients. The purpose of this study is to evaluate the safety and feasibility of different doses of allogeneic BM-MSCs, injected intravenously or intraventricularly in the acute phase of hemorrhagic stroke. No results are presently available (NCT03371329). Therefore, despite the encouraging results described above, further studies are necessary to validate the efficacy of MSC therapy, especially in hemorrhagic stroke.

**Table 3:** Clinical trials using mesenchymal stem/stromal cells in stroke.

Identifier and reference	Recruitment status	MSC source	MSC dose	Delivery route	Main results
**NCT01287936** (Steinberg et al., 2016, 2018)	Completed	Bone marrow (allogeneic—modified to express human Notch-1 intracellular domain—SB623 cells)	Three cohorts: 2.5 × 10^6^, 5.0 × 10^6^, or 10 × 10^6^ cells	Surgical transplantation at residual lesion site	SB623 cells were considered safe and improvement in clinical outcome after 24 months of follow-up was suggested
**NCT01297413** (Levy et al., 2019)	Completed	Bone marrow (allogeneic)	Phase I: 0.5, 1.0, and 1.5 × 10^6^ cells/kg Phase II: 1 × 10^6^ cells/kg	Intravenous	The 1 × 10^6^ cells/kg dose was safe and behavioral improvement was suggested after 12 months of follow-up
**NCT00875654** (Jaillard et al., 2020)	Completed	Bone marrow (autologous)	Two cohorts: 1 × 10^8^ or 3 × 10^8^ cells	Intravenous	Both doses were considered safe and improvement in clinical outcome after 24 months of follow-up was suggested
**NCT03356821**	Completed	Bone marrow (allogeneic)	5 × 10^7^	Intranasal	No results posted
**NCT03371329**	Completed	Bone marrow (allogeneic)	Four cohorts: Cohort 1: 5 × 10^6^ cells/kg Cohort 2: 1 × 10^6^ cells/kg Cohort 3: 2 × 10^6^ cells/kg Cohort 4: 0.5 × 10^6^ cells/kg	Intravenous (cohorts 1, 2 and 3) or Intraventricular (cohort 4)	No results posted
**NCT02378974**	Completed	Umbilical cord (allogeneic)	Two cohorts: Cohort 1: 2 × 10^7^ cells on day 0 Cohort 1: 2 × 10^7^ cells on day 0 and 7	Intravenous	No results posted
**NCT01678534**	Completed	Adipose tissue (allogeneic)	1 × 10^6^ cells	Intravenous	No results posted
**NCT04811651**	Recruiting	Umbilical cord	1 × 10^8^	Intravenous	No results posted
**NCT05158101**	Recruiting	Umbilical Cord	1 × 10^8^	Intravenous	No results posted
**NCT04434768**	Recruiting	Umbilical Cord (allogeneic)	Not available	Combination of intra-arterial intravenous	No results posted
**NCT04280003** (de Celis-Ruiz et al., 2021)	Recruiting	Adipose tissue (allogeneic)	1 × 10^6^ cells/kg	Intravenous	No results posted
**NCT04097652**	Recruiting	Umbilical Cord (allogeneic)	Three cohorts with different cell doses (low, medium, and high—specific values are not mentioned)	Intravenous	No results posted
**NCT03384433**	Recruiting	Exosomes from allogeneic MSCs-enriched by miR-124	Not available	Intra-parenchymal	No results posted
**NCT04953663**	Recruiting	Bone marrow (allogeneic)	Several cohorts, from 0.5–2 × 10^6^ cell/kg	Intravenous	No results posted
**NCT05008588**	Not yet recruiting	Umbilical cord	Cohort 1: 1–2 × 10^6^ UC-MSCs Cohort 2: 1–2 × 10^6^ UC-MSCs + 160 μl MSC-conditioned medium	Intranasal (MSC-conditioned medium) and intra-parenchymal (UC-MSCs)	No results posted
**NCT04590118**	Not yet recruiting	Not available	Different doses ranging from 0.5–2 × 10^6^ cells/kg	Intravenous	No results posted
**NCT04093336**	Recruiting	Umbilical Cord (allogeneic)	2 × 10^6^ cells/kg	Intravenous	No results posted
**NCT01716481** (Lee et al., 2022)	Unknown	Bone Marrow (autologous) expanded with autologous serum from acute phase of stroke	1 × 10^6^ cells	Intravenous	Motor score was significantly higher in the MSC group than in control. Neuroimaging analysis indicate better preservation of corticospinal tract in MSC group.

### Spinal Cord Injury

Spinal cord injury (SCI) is a devastating neurological disorder that compromises sensory, motor, and autonomic functions, resulting in paraplegia or tetraplegia. The pathophysiology of SCI is temporally divided into acute (<48 h), subacute (2–14 days), intermediate (2 weeks to 6 months), and chronic (>6 months) phases and includes a primary injury that usually arises from compression, contusion, or transection of the spinal cord, and a secondary injury cascade that exacerbates the initial damage, causing even greater loss of neurological function (Ahuja et al., 2017). The current treatment for SCI includes surgical decompression, steroid administration, and rehabilitation therapies, which provide poor outcomes. In recent years, MSC transplantation has yielded encouraging results in preclinical and clinical research.

Most preclinical studies use MSCs derived from BM, AT, and UC in acute/subacute SCI models, while few studies have evaluated the efficacy of cell therapy in models of chronic SCI, which is more clinically relevant. Transplantation of BM-MSC sheets in acute and subacute SCI rat models reduced infiltration of inflammatory cells and the area of the injury lesion, promoted axonal regeneration, and attenuated glial scar formation, one of the major limiting factors to functional recovery after SCI (Okuda et al., 2017; Yamazaki et al., 2021). Basso, Beattie, and Bresnahan (BBB) scores showed improved locomotor function in sheet groups. Intravenously or intraperitoneally injected BM-MSCs were able to migrate toward the lesion site, where they survived up to 8 weeks and increased local expression of trophic factors such as BDNF, NGF, and NT3. Better tissue preservation with enhanced fiber sparing and/or regeneration was observed, in addition to the motor and sensory recovery (Ramalho et al., 2018; Dos Santos Ramalho et al., 2019). The benefits of BM-MSCs in acute SCI models were even greater when the cells were transplanted into biomaterial scaffolds. Better motor function recovery, neuroprotection, axonal regeneration, and reduced cavitation were observed 8 weeks after implantation of a nanofibrous silk-fibroin scaffold seeded with 5 × 10^5^ BM-MSCs (Wang X. H. et al., 2021) or an NGF persistent-delivery scaffold seeded with 1 × 10^5^ cells (Ji et al., 2021). Even better outcomes were achieved when BM-MSCs were differentiated *in vitro* into nerve cells prior to transfer onto scaffolds. In a chronic SCI model, intravenous infusion of BM-MSCs promoted neovascularization and improved the integrity of the blood-spinal cord barrier (BSCB). Axonal regeneration and remyelination process were observed, resulting in significant locomotor recovery (Morita et al., 2016).

AT-MSCs also exert positive effects on rats with SCI. AT-MSCs embedded in a fibrin matrix reduced microglial infiltration and astroglial activation as well as upregulated the expression of angiogenic and anti-apoptotic proteins, contributing to less cavitation, tissue retention, and motor-function recovery (Mukhamedshina et al., 2018). Human AT-MSCs transplanted into an acute compressive SCI model induced angiogenesis in the perilesional area and recruited resident pericytes through secretion of paracrine factors, improving BSCB integrity and contributing to the maturation of newly formed blood vessels. Revascularization reduced astrogliosis, promoted neuronal recovery, and correlated with functional improvement (Menezes et al., 2020).

The above-mentioned advantages regarding methods to obtain UC-MSCs and their properties have encouraged their use in the treatment of SCI. In different subacute SCI mouse models, injection of human UC-MSCs into the center of the injury area decreased the local levels of several proinflammatory cytokines such as TNF-α, IFN-γ, IL-6, and IL-7, in addition to promoting the transition of macrophages to the M2 type, attenuating the local inflammatory response and resulting in tissue repair and motor-function recovery (Bao et al., 2018; Wu et al., 2020). Differences in the ability to inhibit the inflammatory response between human UC-MSCs from different donors are determinants of their effectiveness in the recovery of mice after SCI (Zhu et al., 2022). Intrathecal transplantation of human UC-MSCs in rats with subacute SCI promoted regeneration and remyelination, as well as reduced astrogliosis, decreased cavity and glial scar formation, and supported functional recovery (Yang et al., 2020; Cao et al., 2022). The application of WJ-MSCs has also been promising. Intrathecal transplantation of WJ-MSCs following SCI improved neurological function in rats by inhibiting the inflammasome complex (Mohamadi et al., 2019), and their regenerative effect has been shown to be dose-dependent and potentiated by repeated application (Krupa et al., 2018).

In recent years, MSC-derived Evs have been extensively explored as a potential therapeutic strategy for SCI. Both MSC-derived exosomes and small extracellular vesicles are effective in promoting recovery of locomotor function in rodent SCI animal models, mainly by dampening the post-traumatic inflammatory process. The anti-inflammatory effect of BM-MSC-Evs has been shown to be mediated, through different mechanisms, by inhibition of the TLR4/NF-κB signaling pathway (Fan et al., 2021; Nie and Jiang, 2021). Additionally, MSC-Ev infusion promoted macrophage polarization toward the M2-phenotype (Chang et al., 2021; Nakazaki et al., 2021) and improved their phagocytic ability to clear myelin debris, creating a favorable environment for axonal regeneration (Sheng et al., 2021). Greater BSCB integrity was also observed, due to a reduction in pericyte pryoptosis, which was essential for tissue preservation and neuroprotection (Zhou et al., 2022).

Several clinical trials have supported the feasibility and safety of transplantation of MSCs in treating patients with SCI (Satti et al., 2016; Larocca et al., 2017; Albu et al., 2021). However, the trials have not well replicated the motor-function improvement reported in preclinical studies, showing efficacy mainly in sensory recovery. Intrathecal administration of autologous BM-MSCs in patients with SCI promoted variable improvement of sensitivity, motor and sexual functions, spasticity, sphincter control, and urodynamic parameters, regardless of the level or degree of injury (Vaquero et al., 2016, 2017, 2018). Recently, results from a phase 2 study supported the safety of intravenous infusion of autologous BM-MSCs performed in 13 patients approximately 50 days after SCI. The subjects showed improvements in neurological functions and quality of life 6 months after the intervention (Honmou et al., 2021). Combined intrathecal transplantation of these cells with autologous Schwann cells has also promoted improvements, especially in the sensation of bladder and rectal filling (Oraee-Yazdani et al., 2021). Few clinical studies have evaluated the efficacy of AT-MSCs in SCI treatment. Hur and collaborators performed autologous AT-MSC transplantation in patients with complete or incomplete SCI, but only mild improvements in neurological function were achieved (Hur et al., 2016). A preliminary report of an ongoing phase I study conducted at the Mayo Clinic (NCT03308565) using autologous AT-MSCs described promising outcomes and no adverse events (Bydon et al., 2020). Impressive results have been described regarding the efficacy of UC-MSC transplantation in patients with SCI. In a phase I trial with two patients with acute complete injuries, human UC-MSCs loaded on collagen scaffolds (NeuroRegen scaffolds) transplanted into the injury site promoted recovery of the bowel and bladder sensory function and significant motor improvement. Six months post-surgery, one patient started to walk voluntarily with the support of a brace and the other could raise his leg and move his toes (Xiao et al., 2018). The results of long-term follow-up of these and other enrolled subjects showed that three patients with an acute complete injury who received human UC-MSCs recovered the ability to walk and the remaining four patients had significant improvements in sensory and motor functions (Tang et al., 2022). Key completed and ongoing clinical trials of SCI using MSCs are summarized in Table 4.

**Table 4:** Clinical trials using mesenchymal stem/stromal cells in spinal cord injury.

Identifier and reference	Recruitment status	MSCs source	MSCs dose	Delivery route	Main results
NCT02482194 (Satti et al., 2016)	Completed	Bone marrow (autologous)	2 or 3 doses: 1.2 × 10^6^ cells/kg	Intrathecal	Repeated doses were safe with no associated adverse reactions
NCT02152657 (Larocca et al., 2017)	Completed	Bone marrow (autologous)	2 × 10^7^ cells	Percutaneous injection	Intervention was safe and feasible
NCT01909154 (Vaquero et al., 2016)	Completed	Bone marrow (autologous)	100–230 × 10^6^ + 30 × 10^6^ cells 1 month after first injections	Injury site + intrathecal	Dose-dependent improvement of infralesional motor activity, sensation, and sphincter control
NCT02165904 (Vaquero et al., 2017)	Completed	Bone marrow (autologous)	Four doses of 3 × 10^7^ cells	Lumbar puncture	Motor improvement in 60% of cases; improvement in sexual function in 25% of men; 88.8% improvement in bladder function
**NCT02570932** (Vaquero et al., 2018)	Completed	Bone marrow (autologous)	Three doses of 1 × 10^8^ cells	Lumbar puncture	Improvement of urodynamic parameters in 66.6% of patients; 55.5% improvement in somatosensory or motor-evoked potentials; 44.4% improvement in voluntary muscle contraction
**NCT02981576**	Completed	Bone marrowvs Adipose tissue (autologous)	Three doses of BM-MSCs or AT-MSC values not available	Intrathecal	No results posted
**NCT04288934**	Completed	Bone marrow (autologous) vs. Wharton’s Jelly (allogeneic)	Not available	Intraspinal	No results posted
**NCT02352077**	Unknown	Bone marrow + NeuroRegenscaffold	Not available	Injury site	No results posted
**NCT02574585**	Unknown	Bone marrow (autologous)	Not available	Percutaneousinjections	No results posted
**NCT02574572**	Unknown	Bone marrow (autologous)	Not available	Intralesional	No results posted
**(Hur et al., 2016)**	Completed	Adipose tissue (autologous)	9 × 10^7^cells	Intrathecal	Mild improvements in neurological function
**NCT01769872**	Completed	Adipose tissue(autologous)	2 × 10^8^ cells/20 ml 5 × 10^7^ cells/2 ml 2 × 10^7^ cells/1 ml	Intravenous Intrathecal Intraspinal	No results posted
**NCT02917291**	Recruiting	Adipose tissue (FAB117-HC)(allogeneic)	Phase 1: Two cohorts: 2 × 10^7^ or 4 × 10^7^ cells Phase 2: administration of the maximum tolerated dose in phase 1	Intramedullary	No results posted
**NCT04520373**	Recruiting	Adipose tissue (autologous)	Not available	Intrathecal	No results posted
**NCT03308565** (Bydon et al., 2020)	Active, not recruiting	Adipose tissue (autologous)	1 × 10^8^ cells	Intrathecal	Administration and dose were safe and feasible with meaningful signs of improved neurologic status
**NCT01873547**	Completed	Umbilical Cord (allogeneic)	Not available	Lumbar puncture	No results posted
**NCT02481440** (Yang et al., 2021)	Completed	Umbilical cord (allogeneic)	Four doses of 1 × 10^6^ cells/kg	Intrathecal	Intervention was safe with any adverse events and improved scores on sensory, motor, and functional assessment scales and in bladder and bowel function
**NCT03003364** (Albu et al., 2021)	Completed	Wharton’s Jelly (allogeneic)	1 × 10^7^cells	Intrathecal	Sensory improvement in the segments adjacent to the injury site
**NCT04213131**	Unknown	Umbilical cord and umbilical cord blood (allogeneic)	5 × 10^7^ cells/100 ml 5 × 10^7^ cells/5 ml 1 × 10^5^ cells/μl, 16 μl/point (4 points)	Intravenous Lumbar Injury site	No results posted
**NCT03521323** **NCT03521336** **NCT03505034**	Unknown	Umbilical cord (allogeneic)	Four doses of 1 × 10^6^ cells/kg	Intrathecal	No results posted
**NCT02510365** (Xiao et al., 2018)	Unknown	Umbilical cord + NeuroRegen Scaffolds (allogeneic)	4 × 10^7^cells	Injury site	One patient was able to walk voluntarily under the help of brace and the other was able to raise the leg and move toes
**NCT02688049**	Unknown	NeuroRegen scaffold/MSC	1 × 10^7^ cells	Intraspinal	No results posted

### Autism

Autism was first described in 1930 as a clinical disorder that affects social interaction and communication (Kanner, 1943). Currently, it comprises a series of disorders that are described as Autistic Spectrum Disorders (ASD). ASDs encompass a spectrum of neurological disorders related to an abnormal neurodevelopment process. These disorders affect individuals early in life, with clinical symptoms manifesting until the age of 5 years (Lord et al., 2006). The diagnosis, which is performed by clinical evaluation, follows international standards determined by the standards described in DSM-5 (Diagnostic and Statistical Manual of Mental Disorders) and takes into account deficiencies in social interaction, communication, limited interest, and repetitive behavior (Lord et al., 2006).

According to the Autism and Developmental Disabilities Monitoring (ADDM) Network and the Centers for Disease Control and Prevention (CDC), responsible for surveillance of ASD in the United States, about 1 in 54 children has an autism spectrum disorder, with an approximately fourfold higher incidence in males (Maenner et al., 2020). The number of patients diagnosed with autism has increased exponentially, which may be related both to an increase in the number of affected individuals and to an increase in previously undetermined diagnoses that are now included within the ASDs.

As ASDs include a diversity of disorders, there is wide heterogeneity both in the severity of symptoms and in the molecular mechanisms that lead to the development of ASD (Geschwind and Levitt, 2007). In most cases, ASDs are associated with genetic factors; more than 100 conditions have been directly associated with these disorders, with dozens of genes described as susceptibility factors (Betancur, 2011). The genetic changes described so far are related to transcriptional control, chromatin remodeling, protein synthesis, cell metabolism, function, and development of synapses (Thapar and Rutter, 2021). Several environmental risk factors are also involved in the development of ASD, and the complex interaction between genetic and environmental factors might determine the impact on neurodevelopment and the severity of neural-cognitive impairments. Among the physiological impairments, a critical one is neuroinflammation (Bilbo et al., 2018), and abnormal activation of microglia has been reported in the brain of ASD patients (Vargas et al., 2005; Morgan et al., 2012; Lee et al., 2017). While in a healthy environment, microglia are associated with synaptic organization and brain repair, when abnormally activated, microglia leads to inflammation and damage (Conti et al., 2020), as observed in degenerative neurological diseases such as Alzheimer’s (Wang et al., 2015) and Traumatic Brain Injury (Hernandez-Ontiveros et al., 2013). Other than microglia, immune system molecules such as TGFβ, IL-1, and LIF are important for brain development because they are directly involved in the normal formation of synaptic neural circuits and cell differentiation, and the imbalance in the expressions of these molecules may lead to devastating consequences (Deverman and Patterson, 2009).

Currently, none of the diseases within the spectrum has a cure; treatments include therapies to assist the interaction of these individuals with society, behavioral therapies, and the use of drugs to treat associated symptoms such as seizures (Eissa et al., 2018). However, the role of inflammation in some of the disorders in the autistic spectrum opens a possibility for the use of immunomodulation as a strategy for the development of new therapies (Marchezan et al., 2018). In light of this, MSCs emerge as a promising tool, since these cells have the ability to migrate to inflammation sites and modulate the immune response through the paracrine secretion of several anti-inflammatory, pro-survival, and neurotrophic factors, and are also able to engraft into the neural network (Siniscalco et al., 2018). Animal models available for studies of ASD include BTBR T+tf/J, a mouse strain that shows impairment in communication, deficits in social interaction, repetitive behaviors, and anxiety, mimicking the main symptoms observed in ASD patients (Chao et al., 2018). Studies with these mice suggested an improvement in neurogenesis, specifically in the hippocampal area, and in social behavior in animals treated with MSCs, where an amelioration of repetitive behavior, a decrease in cognitive rigidity, and in the autism severity composite score as a whole were observed (Segal-Gavish et al., 2016). Another study with MSCs pretreated with hbFGF, PDGF-AA, and Heregulin β1 to induce higher secretion of neurotrophic factors, also found an improvement in mouse social behavior, communication through vocalization, and reduction in repetitive behavior and cognitive rigidity (Perets et al., 2017).

MSCs were rapidly included in clinical studies of ASD. To date, three studies conducted in three different countries have been completed, and demonstrated the safety of using MSCs in ASD patients (Table 5). The preferred source of MSCs in the ASD clinical trials was UC since these cells are weakly immunogenic and powerfully immunosuppressive (Nagamura-Inoue and He, 2014). The first clinical study, published in 2013 (Lv et al., 2013) performed a trial using mononuclear cells and UC-MSCs. They separated 37 patients into three groups: 14 received only mononuclear cells, nine received both mononuclear and UC-MSCs, and 14 served as a control group; all groups received rehabilitation therapy. The study found functional and subjective improvements in patients of both groups treated with cells, assessed through the change in scores on the Childhood Autism Rating Scale (CARS) and Aberrant Behavior Checklist (ABC). In 2019, another group performed a phase I-II clinical trial using only UC-MSCs to treat ASD patients and evaluated the effects on cytokine levels (Riordan et al., 2019). Twenty participants (children around 10 years of age) received four intravenous infusions of 9 × 10^6^ cells over the course of 1 week, three times (infusions in weeks 1, 25, and 37), and were followed for up to 89 weeks after the first injection. The investigators opted for multiple UC-MSC injections based on their clinical observations that the effect of MSC infusion decays 3–6 months after the cell administration. The authors observed this tendency even after multiple injections in a group of patients, who showed slight improvement but then regressed to the initial scores in CARS. Another set of patients, however, was able to sustain the improvement in test scores, in both behavioral (CARS) and cytokine scores [macrophage-derived chemokine (MDC) and thymus, and activation-regulated chemokine (TARC)]. However, the positive results for this small group were not statistically significant. Nevertheless, this study helped to support the safety of repeated, periodic administration of UC-MSCs in a long-term treatment in children with ASD (this article was retracted due to ethical/financial issues, but the clinical trial data are valid and available). The third and most recently published clinical trial with UC-MSCs in ASD is a phase I clinical study with cord-tissue mesenchymal stromal cells (hCT-MSC) administered intravenously, 2 × 10^6^ cells/kg, to 12 young ASD children (2–11 years old; Sun et al., 2020). This study did not find an improvement in any behavioral parameter, but focused on the safety of the treatment, showing that HLA antibodies were generated against some of the cell lines, suggesting that it is important to evaluate if this could affect the treatment. Despite the presence of anti-HLA antibodies, the study demonstrated the safety of transplanting hCT-MSCs. This group has planned three additional clinical studies to investigate the effect of hCT-MSC transplantation in ASD adults (18–35 years of age; NCT04484077), toddlers (18–48 months; NCT04294290), and a larger study with 164 children (4–11 years), presently in the recruitment stage (NCT04089579).

**Table 5:** Clinical trials using mesenchymal stem/stromal cells in autism spectrum disorder.

Identifier and reference	Recruitment status	MSC source	MSC dose	Delivery route	Main results
**NCT02192749**	Completed	Umbilical cord	9 × 10^6^ cells/infusion (four infusions)	Intravenous	UC-MSC infusions were safe and tolerable
**NCT03099239**	Completed	Umbilical cord	2 × 10^6^ cells/kg	Intravenous	Overall, infusions were safe and well tolerated
**NCT01343511**	Completed	Mononuclear and Umbilical cord	2 × 10^6^ cells/kg CBMNCs and 1 × 10^6^ cells/kg UC-MSCs	Intravenous and/or intrathecal	There were no significant safety issues related to the treatment and no observed severe adverse effects
**NCT04089579**	Recruiting	Umbilical cord	6 × 10^6^ cells/kg	Intravenous	No results posted
**NCT04484077**	not yet recruiting	Umbilical cord	2 × 10^6^cells/kg (max 10 × 10^7^)	Intravenous	No results posted
**NCT04294290**	not yet recruiting	Umbilical cord	2 × 10^6^/kg	Intravenous	No results posted

Clinical trials have so far shown the safety of using MSCs in ASD patients, and a promising, but still incipient, the potential for improving the condition of the patients. Another aspect of these studies is the enormous quantity of cells that are necessarily specific for each patient, which could limit the widespread use of the therapy. Considering those aspects, new preclinical studies have shown that ASD individuals could benefit from the MSC effects without the need to manufacture such a large quantity of cells from different donors, or other issues such as immunogenicity and tumor formation, by using exosomes instead. Exosomes might be isolated from the expansion of a single donor MSC and used allogeneically to treat multiple patients (Mendt et al., 2019). The Perets group, who demonstrated the efficacy of BM-MSCs to improve autistic-like behavior in BTBR T+tf/J (BTBR) mice in 2017, has shown that administration of MSC-exosomes had similar effects, increasing male-to-male social interaction and reducing repetitive behaviors, as well as female vocalizations and maternal behaviors (Perets et al., 2018). Moreover, exosomes could be administered as a non-invasive intranasal treatment, loaded with gold nanoparticles that are able to cross the blood-brain barrier and preserve all their properties; this would make the treatment appealing commercially and clinically, especially considering the unstable behavior of ASD patients (Geffen et al., 2020; Perets et al., 2020). Independently, another group performed a similar experiment, with intranasal administration of exosomes, and demonstrated improvement in mouse social behavior; reduction of the pro-inflammatory cytokines IL-1b, IL-6, and TNFα; and increase of the anti-inflammatory cytokine IL-10 in the brains of treated mice, suggesting that exosomes have anti-inflammatory and neuroprotective roles in the autistic brain (Liang et al., 2020). Further studies are required to evaluate the efficacy of MSC exosome therapy and unravel the molecular pathways involved in the effector mechanisms.

Additional information regarding current MSC-based clinical trials in other neurological disorders such as multiple sclerosis, Alzheimer’s, Parkinson’s, and Huntington’s diseases is summarized in Table 6. All clinical trials reported here were accessed on clinicaltrial.gov between January and March 2022.

**Table 6:** Clinical trials using mesenchymal stem/stromal cells in other neurological disorders.

Condition	Identifier and reference	Recruitment status	MSC source	MSC dose	Delivery route	Main results
**Huntington disease**	NCT02728115 (SAVE-DH Study—Phase I)	Active, not recruiting	Not available	Cohort 1: 1 × 10^6^ cells/kg Cohort 2: 2 × 10^6^ cells/kg (Both cohorts receive three injections—1 each month for 3 months)	Intravenous	No results posted
**Huntington disease**	NCT03252535 (ADORE-DH Study—Phase II)	Complete	Not available	Cohort 1: 1 × 10^6^ cells/kg Cohort 2: 2 × 10^6^ cells/kg (Both cohorts receive nine injections, divided in three cycles)	Intravenous	No results posted
**Huntington disease**	NCT04219241 (ADORE-EXT Study—Phase II/III)	Not yet recruiting	Not available	2 × 10^6^ cells/kg (12 injections—three administrations per cycle). Each administration will occur every 30 days and cycles every 180 days (total of four cycles)	Intravenous	No results posted
**Multiple sclerosis**	NCT01730547 NCT01854957 NCT02403947 NCT01745783 NCT01606215 NCT02035514 NCT02239393 (Uccelli et al., 2019)	Unknown Unknown Terminated Active, not recruiting Completed Completed Completed	Bone marrow (autologous)	1–2 × 10^6^ cells/kg	Intravenous	No results posted
**Multiple sclerosis**	NCT02326935	Terminated	Adipose tissue (autologous)	1.5 × 10^8^ cells	Intravenous	No results posted
**Multiple sclerosis**	NCT02034188 (Riordan et al., 2018)	Completed	Umbilical cord	2 × 10^7^ cells	Intravenous	Therapy was safe and well-tolerated. Symptom improvements observed 1 month after treatment
**Multiple sclerosis**	NCT01228266 (Llufriu et al., 2014)	Terminated	Bone marrow	1–2 × 10^6^cells	Intravenous	Therapy was safe and well-tolerated. Tendency to reduce inflammation parameters. No significant differences detected in the secondary endpoints.
**Multiple sclerosis**	NCT00395200 (Connick et al., 2011, 2012)	Completed	Bone marrow (autologous)	1.1–2 × 10^6^cells/kg	Intravenous	Therapy was safe and well-tolerated. Visual acuity and visual evoked response latency improvement.
**Multiple sclerosis**	NCT03326505 (Alghwiri et al., 2020)	Completed	Wharton’s Jelly	1 × 10^8^ cells (IT) and 5 × 10^7^ (IV)— 2×, 1 month apart Conditioned medium (IT–Third month)	Intrathecal Intravenous	No results posted
**Multiple sclerosis**	NCT01933802 NCT03355365 (Harris et al., 2018)	Completed Active, not recruiting	Bone marrow mesenchymal stem cell-derived neural progenitor (autologous)	1 × 10^7^ cells per dose (3×, 1 month apart)	Intrathecal	Therapy was safe and well-tolerated.
**Multiple sclerosis**	NCT00813969 (Cohen et al., 2018)	Completed	Bone marrow (autologous)	1–2 × 10^6^ cells/kg	Intravenous	Therapy was safe and well-tolerated.
**Multiple sclerosis**	NCT04749667	Recruiting	Bone marrow (autologous)	Not available	Intrathecal	No results posted
**Multiple sclerosis**	NCT04823000 (Petrou et al., 2021b)	Completed	Bone marrow (autologous)	Multiple injections (up to eight courses) of 1 × 10^6^ cells/kg	Intrathecal and intravenous	Therapy was safe and well-tolerated. Indication of clinical benefits.
**Multiple sclerosis**	NCT02495766	Completed	Bone marrow (autologous)	Not available	Not available	No results posted
**Multiple sclerosis**	NCT00781872 (Karussis et al., 2010)	Completed	Bone marrow (autologous)	6 × 10^7^ cells intrathecal and 2 × 10^7^ cells intravenous	Intrathecal and Intravenous	Therapy was safe and well-tolerated.
**Multiple sclerosis**	NCT05003388	Recruiting	Umbilical cord	1 × 10^8^ cells	Intravenous	No results posted
**Multiple sclerosis**	NCT05116540	Recruiting	Adipose tissue (autologous)	Six infusions Dose not available	intravenous	No results posted
**Multiple sclerosis**	NCT03822858	Temporarily not available	Bone marrow (autologous)	Not available	Intrathecal	No results posted
**Multiple sclerosis**	NCT03799718	Completed	Bone marrow secreting neurotrophic factors– MSC-NTF, NurOwn^TM^ (Autologous)	Not available	Intrathecal	No results posted
**Alzheimer’s disease**	NCT02600130	Completed	Bone marrow (Longeveron)	Two cohorts: 2 × 10^7^ or 1 × 10^8^ cells	Intravenous	No results posted
**Alzheimer’s disease**	NCT02833792	Recruiting	Bone marrow Stemedica (allogeneic)	1.5 × 10^6^ cells/kg+ Lactated Ringer’s Solution	Intravenous	No results posted
**Alzheimer’s disease**	NCT03117738	Completed	Adipose tissue (autologous)	Not available	Intravenous	Treatment related adverse events in 54.5% of subjects
**Alzheimer’s disease**	NCT04388982	Recruiting	Adipose tissue exosomes (allogeneic)	Three cohorts: 5 μg MSC-Exos/1 ml, 10 μg MSC-Exos/1 ml or 20 μg MSC-Exos/1 ml twice a week for 12 weeks	Nasal drip	No results posted
**Alzheimer’s disease**	NCT04482413	Not yet recruiting	Adipose tissue (autologous)	Two doses of 2 × 10^8^/20 ml every 4 weeks from week 0 to week 16	Intravenous	No results posted
**Alzheimer’s disease**	NCT02054208 (Kim H. J. et al., 2021)	Completed	Umbilical cord blood–NEUROSTEM^®^ (allogeneic)	Three doses of 1 × 10^7^ cells/2 ml or Three doses of 3 × 10^7^ cells/2 ml	Intraventricular (Ommaya Reservoir)	Interventions were feasible, relatively and sufficiently safe, and well-tolerated
**Alzheimer’s disease**	NCT03172117 (Kim H. J. et al., 2021)	Recruiting	Umbilical cord blood NEUROSTEM^®^ (allogeneic)	Two cohorts: Three doses of 1 × 10^7^ cells/2 ml or Three doses of 3 × 10^7^ cells/2 ml	Intraventricular (Ommaya Reservoir)	Interventions were feasible, relatively and sufficiently safe, and well-tolerated
**Alzheimer’s disease**	NCT04954534	Not yet recruiting	Umbilical cord blood NEUROSTEM^®^ (allogeneic)	Three doses of 3 × 10^7^ cells/2 ml	Intraventricular (Ommaya Reservoir)	No results posted
**Alzheimer’s disease**	NCT04040348	Active, not recruiting	Umbilical cord (allogeneic)	Four doses of 1 × 10^8^ cells	Intravenous	No results posted
**Alzheimer’s disease**	NCT02672306	Unknown	Umbilical cord (allogeneic)	Eight doses of 0.5 × 10^6^ cells/kg	Intravenous	No results posted
**Alzheimer’s disease**	NCT02899091	Unknown	Placenta CB-AC-02 (allogeneic)	Stage 1: 1 or Two doses of 2 × 10^8^ cells Stage 2: Two doses of 2 × 10^8^ cells	Intravenous	No results posted
**Alzheimer’s disease**	NCT04684602	Recruiting	Amnion and umbilical cord (allogeneic)	Not available	Not available	No results posted
**Parkinson’s disease**	NCT04876326	Recruiting	Adipose tissue Umbilical cord	Two doses of 5 × 10^7^	Intrathecal	No results posted
**Parkinson’s disease**	NCT01824121 (Giordano et al., 2014; Canesi et al., 2016; Giordano et al., 2021)	Recruiting/Unkown	Bone marrow	1.2–2.0 × 10^6^ cells/kg	Intra-arterial	The overall safety and efficacy results are still inconclusive, because of the low number of patients and consequently the poor statistical power of the study.
**Parkinson’s disease**	NCT03550183	Enrolling by invitation	Umbilical cord	1–2 × 10^7^ cells	Intravenous	No results posted
**Parkinson’s disease**	NCT01446614 (Zhang et al., 2008)	Recruiting/Unknown	Bone marrow	6 × 10^5^ cells/kg	Intravenous	MSCs derived from PD patients’ bone marrow may be a promising cell type for cellular therapy
**Parkinson’s disease**	NCT04506073	Active, not recruiting	Bone marrow	Two doses of 1 × 10^7^ cell/kg	Not available	No results posted
**Parkinson’s disease**	NCT04146519 (Boika et al., 2020)	Recruiting	Bone marrow	0.5–2 × 10^6^ cells/kg	Intravenous Intranasal	Decrease in the severity of motor and nonmotor symptoms
**Parkinson’s disease**	NCT05152394	Not yet recruiting	Umbilical cord	1 × 10^8^ cells	Intravenous	No results posted
**Parkinson’s disease**	NCT04064983	No longer available	Adipose tissue	2 × 10^8^ cells	Intravenous	No results posted
**Parkinson’s disease**	NCT05094011	Not yet recruiting	Adipose tissue	1 × 10^8^ cells/hemisphere	Intrastriatal	No results posted
**Parkinson’s disease**	NCT03684122 (Jamali et al., 2021)	Active, not recruiting	Umbilical cord	80–120 × 10^6^ cells	Intrathecal Intravenous	Study chart implementation, data collection, and analysis are ongoing.
**Parkinson’s disease**	NCT00976430	Terminated	Bone marrow	Not available	Not available	No results posted
**Parkinson’s disease**	NCT04928287	Active, not recruiting	Adipose tissue	Not available	Intravenous	No results posted
**Parkinson’s disease**	NCT04995081	Recruiting	Adipose tissue	Not available	Intravenous	No results posted
**Parkinson’s disease**	NCT04772378	No longer available	Adipose tissue	2 × 10^8^ cells	Not available	No results posted
**Parkinson’s disease**	NCT04798066	Available	Adipose tissue	2 × 10^8^ cells	Intravenous	No results posted

## Quality Control of MSCs for Neurological Diseases

Quality control refers to examining whether the characteristics of a product meet a series of pre-specified criteria. Critical quality attributes such as differentiation, proliferation, genomic stability, sterility, and functionality depend on the cell type, sources of origin, and the purpose of the research. Quality controls for MSC culturing and release testing must be appropriately quantified and characterized, to ensure that the manufacturing process is robust and consistently produces MSCs with identical properties from one batch to the next.

With several clinical trials involving MSCs currently underway, there is a critical need to develop standards that can be applied to processing methods and to establish a consensus on assays for both MSC processing control and MSC product release.

Since the advent of clinical translation of MSCs, assays, and test protocols have been required by regulatory agencies to evaluate the sterility, safety, viability, identity, purity, stability, and potency of the cell product to be administered to patients. Additionally, GMP is concerned with both production and quality control. The U.S. Food and Drug Administration (FDA) and the European Medicines Agency (EMA) have similar requirements. As defined, GMP guidelines cover not only the actual physical process of making the cell product but also the quality assurance that the product is produced under conditions that are consistent, safe, and effective for their intended use.

In the United States, guidelines for cell-based therapeutics are regulated by the FDA (U. S. Department of Health and Human Services Food and Drug Administration, Center for Biologics Evaluation and Research, 2001) and are included in the drug manufacturing regulations as described in Title 21 of the Code of Federal Regulations (CFR), including the use of human tissue and cell products (21CFR1271). In Europe, the European Medicines Agency (EMA) produces the Guideline on Human Cell-based Medicinal Products (EMEA/CHMP/410869/2006—European Medicines Agency, 2008). The same approach has been adopted by MHRA in the UK (Medicines and Healthcare Products Regulatory Agency, 2015). According to these regulatory bodies, hMSCs should be characterized based on their most critical attributes, according to the legal requirements previously established by their guidelines and which are summarized in Table 7.

**Table 7:** Release specifications for hMSC-based products according to GMP standards of the two major regulatory authorities.

Parameter	Assays	Acceptance criteria	Regulatory frames	
			FDA	EMA/MHRA
Cell identity				
Phenotype	Flow cytometry	Identification of markers depending on the cell population and origin	21 CFR 610.14	Guideline on Human Cell-Based Medicinal Products (May, 2008) 4.2.3
Cell purity	Indication and quantity of unwanted cells
Impurities				
Adventitious viruses	*In-vitro* adventitious viral agent test	Negative	USP <1050.1> Practical Approaches to ICH Q5A	Guideline on Human Cell-Based Medicinal Products (May, 2008) 4.2.3
Viability				
Viable cells	Living/dead cell count	>70% (FDA) >80% (EMA)	USP <1046> Cell and Gene Therapy Products	Eur. Ph. (2.7.29.) Nucleated cell count and viability
Potency				
Biological activity	ELISA (as possible example of potency)	Specific for cytokine	21.CFR 610.10	Guideline on Human Cell-Based Medicinal Products (May 2008) 4.2.3
Microbiological control				
Sterility	Direct inoculation	No growth	–21 CFR 610.12 Sterility –USP <71> Sterility	– Eur. Ph.: (2.6.27) Microbiological control of cellular products –Eur. Ph.: (2.6.1.) Sterility
Endotoxin detection	LAL test	No detection	– USP: <85> Bacterial Endotoxins Test, USP 33 Reissue –21 CFR 600.3 –21 CFR 610.9	Eur. Ph. (2.6.14.) Monograph on Bacterial endotoxins
Mycoplasma test	PCR	Negative	USP <63> Mycoplasma Tests	Eur. Ph. (2.6.7.) Monograph Mycoplasmas

*Abbreviations: LAL, *Limulus* amebocyte lysate; ELISA, enzyme-linked immunosorbent assay; Eur. Ph., European Pharmacopoeia; FDA, Food and Drug Administration; EMA, European Medicines Agency; USP, United States Pharmacopeia; PCR, polymerase chain reaction*.

For each step in a process, whether it involves cell isolation or enrichment, *in vitro* culture, genetic modification, or final product fill-and-finish, the overall approach should be to reduce the risk of contamination of the product, establish documentation to verify that the entire process is correctly performed, and minimize variability in the process while maintaining the salient characteristics and function of the cells of interest. To demonstrate process control and monitor variability, assays should be developed to determine the cell phenotype, genotype, and/or function at the critical steps of the process. The final product should be tested for identity, safety (viral), purity, potency, sterility, endotoxin, and mycoplasma.

To date, one MSC-based product has been approved for neurological diseases. This is Lenzumestrocel (Neuronata-R^®^ Inj), produced by Corestem Inc., a SouthKorean biotech company. Corestem launched Lenzumestrocel for amyotrophic lateral sclerosis, and the product was approved as an orphan drug for the treatment of ALS by the Ministry of Food and Drug Safety (MFDS) in South Korea in 2014. Lenzumestrocel is based on autologous BM-derived MSCs that are isolated and mixed with cerebrospinal fluid collected from the patient, to be administered as the final product by intrathecal injection. The first Lenzumestrocel injection takes place 4 weeks after the first bone-marrow extraction, followed by a second injection 4 weeks later. The proposed mechanism of action is based on several effects that can prevent motor-neuron death and slow the progression of ALS, such as an action on regulatory T lymphocytes, a neuroprotective effect by the expression of growth factors, and an anti-inflammatory effect due to microglial cells (Oh et al., 2015, 2018). More specifically, for Lenzumestrocel release, the manufacturers adopted the following criteria: viability (94%), sterility (0% contaminants), identity (CD29, CD44, CD73, CD105, CD34, and CD45), and as potency, the level of VEGF measured in pg per 104 cells (Oh et al., 2018). These criteria are not dissimilar to the requirements within the FDA and EMA regulatory frames.

For MSC-based products, cell sources and isolation processes vary widely, and it is best to address any concerns early in development. Among the available assays to characterize MSCs, cell phenotyping by multiparametric flow cytometry has been most often used to identify and enumerate cell subsets (Robb et al., 2019).

While a consensus on MSC identity has matured, it remains challenging to establish assays to measure and predict the mechanisms of action of MSCs in neurological disorders, the so-called potency assay. This is the measure of activity using a suitably quantitative biological assay and is based on the product attribute(s) associated with the relevant biological properties. A correlation between the expected clinical response and the activity in the biological assay should be established in pharmacodynamic or clinical studies (Rudge and Nims, 2017). While mandatory for more-advanced phase III studies, this is an aspect to consider during early clinical investigations (phase I/II).

The definition of potency particularly applied to MSCs for neurological diseases, is a critical aspect of quality testing (Bravery et al., 2013; Galipeau et al., 2016). Several aspects must be considered, starting from the likelihood that a single assay may not provide an adequate definition of MSC potency for neurological diseases where cells may have complex mechanisms of action. In addition, MSCs can have multiple active ingredients and/or multiple biological activities, which may be influenced by host-related microenvironmental signals. Finally, a single biological assay may not be quantitative or be insufficiently robust to define the properties of MSCs. Therefore, investigators should make efforts to translate the biological hypothesis, driving early research on assays that may quantify, for example, MSC pro-angiogenic, anti-apoptotic, or anti-inflammatory properties (Jiao et al., 2011) to be progressively validated within preclinical *in vivo* and clinical developments.

While protocols for quality testing of MSC products are now available, such as immunophenotyping, microbial sterility, endotoxin, and mycoplasma testing, and karyology, better-defined tests of potency and clinical efficacy are required. This is a concerning aspect in the cell-therapy field, calling for the development of international standards. This endeavor will not only benefit all discoveries related to MSC biology but will firmly establish the promising MSC therapeutic profile for still-lethal neurological diseases.

## Conclusion

MSC-based cell therapies are among the strategies most often used for the treatment of neurological disorders in preclinical studies, because of the several advantages provided by these cells, such as an increase in cell survival and proliferation, neuroprotection and regeneration, immunomodulation, as well as a delay of disease progression in some cases. Additionally, the potential of MSCs could be enhanced by genetic modification of these cells, aiming toward overexpression of factors involved in cellular regeneration, or by pre-treatment of MSCs under different culture conditions. Besides, due to their secretory capacity, cell-free approaches using MSC-derived exosomes and extracellular vesicles emerge as another possible strategy. In addition, although some results are not entirely promising, clinical studies have also shown positive effects such as safety, tolerability, and functional improvements after transplantation of MSCs. However, further studies might aid in developing better strategies to obtain larger quantities of healthy cells for use in cell therapies and to reduce the variability of results due to the biological characteristics of MSCs.

## Author Contributions

MS, MD, and RM-O conceived the main topic ideas and planned the manuscript. RG, JV, GLAS, FG, TS, AS-J, GS, and DS wrote subsections of the manuscript and prepared tables. RG edited the manuscript. JV prepared the figure. All authors contributed to the article and approved the submitted version.

## Conflict of Interest

The authors declare that the research was conducted in the absence of any commercial or financial relationships that could be construed as a potential conflict of interest.

## Publisher’s Note

All claims expressed in this article are solely those of the authors and do not necessarily represent those of their affiliated organizations, or those of the publisher, the editors and the reviewers. Any product that may be evaluated in this article, or claim that may be made by its manufacturer, is not guaranteed or endorsed by the publisher.
